# Proteomic profiling of centrosomes across multiple mammalian cell and tissue types by an affinity capture method

**DOI:** 10.1016/j.devcel.2023.09.008

**Published:** 2023-10-17

**Authors:** Sarah Carden, Elisa Vitiello, Ivan Rosa e Silva, James Holder, Valentina Quarantotti, Kamal Kishore, Valar Nila Roamio Franklin, Clive D’Santos, Takashi Ochi, Mark van Breugel, Fanni Gergely

**Affiliations:** 1 CRUK Cambridge Institute, Li Ka Shing Centre, University of Cambridge, Cambridge, UK; 2 Department of Biochemistry, University of Oxford, Oxford, UK; 3 MRC Laboratory of Molecular Biology, Cambridge, UK; 4 The Astbury Centre for Structural Molecular Biology, School of Molecular and Cellular Biology, University of Leeds, Leeds, UK; 5 School of Biological & Behavioural Sciences, Queen Mary University of London, London, UK

## Abstract

Centrosomes are the major microtubule-organizing centers in animals and play fundamental roles in many cellular processes. Understanding how their composition varies across diverse cell types and how it is altered in disease are major unresolved questions, yet currently available centrosome isolation protocols are cumbersome and time-consuming, and they lack scalability. Here, we report the development of centro-some affinity capture (CAPture)-mass spectrometry (MS), a powerful one-step purification method to obtain high-resolution centrosome proteomes from mammalian cells. Utilizing a synthetic peptide derived from CCDC61 protein, CAPture specifically isolates intact centrosomes. Importantly, as a bead-based affinity method, it enables rapid sample processing and multiplexing unlike conventional approaches. Our study demonstrates the power of CAPture-MS to elucidate cell-type-dependent heterogeneity in centrosome composition, dissect hierarchical interactions, and identify previously unknown centrosome components. Overall, CAPture-MS represents a transformative tool to unveil temporal, regulatory, cell-type- and tissue-specific changes in centrosome proteomes in health and disease.

## Graphical abstract

**Graphical abstract:**
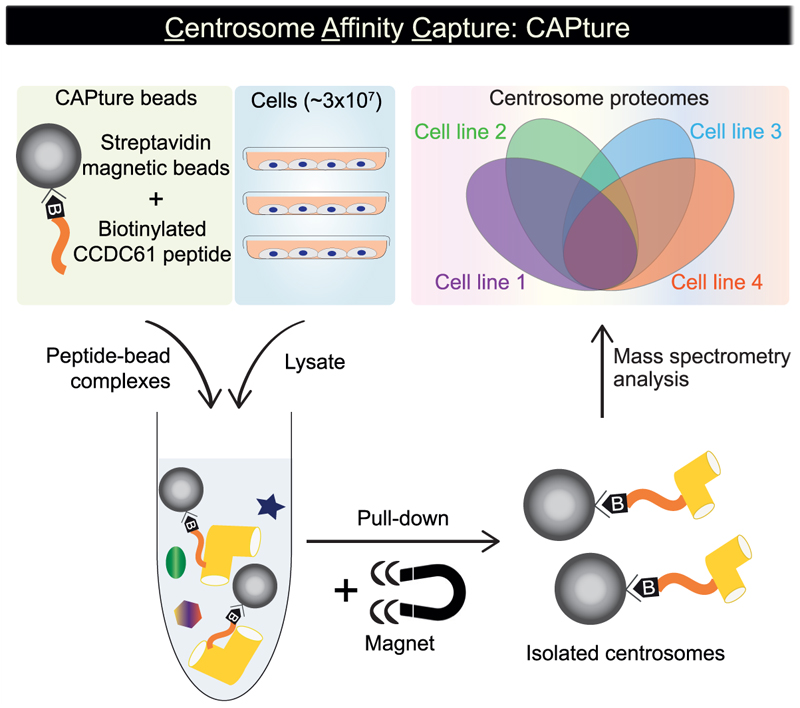

## Introduction

Centrosomes are membraneless organelles that comprise a pericentriolar matrix (PCM) and a pair of cylindrical centrioles.^1,2^ A tightly controlled duplication cycle ensures that cells have the correct number of centrosomes. Cells are born with a daughter and mother centriole, each of which templates assembly of a single procentriole. Briefly, in S-phase centrosomal scaffold proteins drive accumulation of polo-like kinase 4 (PLK4), the master kinase of centrosome duplication, at the proximal ends of parental centrioles.^3–5^ PLK4 recruits SCL/TAL interrupting locus (STIL) that in turn stabilizes the kinase, leading to recruitment of spindle assembly abnormal protein 6 (SAS-6), the key component of the cart-wheel scaffold.^6–9^ Microtubule blades then assemble around the cartwheel to form the centriole wall and elongate through S- and G_2_-phases.^10–14^ Each procentriole remains tightly associated with its parent until late mitosis, when disengagement triggers conversion of procentrioles into daughter centrioles by enabling PCM recruitment.^15^ At the periphery of the PCM, spherical granules, called centriolar satellites, mediate intra-cellular signaling and protein degradation.^16–18^ In many cell types, the mother centriole gets converted into a basal body, which templates formation of a cilium, a sensory and signaling organelle. Distal appendages (DAPs), a structure unique to mother centrioles, provide a platform for early steps of cilia formation and are therefore essential for ciliogenesis.^16^ Mother centrioles also bear subdistal appendages (sDAPs) implicated in microtubule anchoring and centrosome/cilia positioning.^19^ Daughter centrioles mature into mothers by acquiring sDAPs and DAPs during G2-phase.^2^

Proteomic profiling of microtubule-organizing centers was performed first on yeast spindle pole bodies,^20^ followed by studies on human centrosomes by Andersen and colleagues.^21^ Briefly, using quantitative mass spectrometry (MS) methods, they analyzed centrosomes enriched by sucrose density gradient (SDG) centrifugation^22^ from KE-37 leukemia cells, ultimately identifying ~160 centrosomal proteins.^23,24^ More recently, targeted proteomics revealed the absolute quantities of select proteins at the centrosome.^25^ While SDG centrifugation can enrich centrosomes for MS analysis, preparations are inherently impure due to co-sedimentation of centrosomes with other organelles and protein complexes. Furthermore, SDG centrifugation is labor intensive and requires large cell numbers, thereby limiting its use across cell types. Alongside proteomic studies, valuable insight into centrosome substructures have been gained by imaging methods including electron, confocal, and super-resolution microscopy, and ultrastructure expansion microscopy (U-ExM).^2,26^

Spatial proteomic profiling by proximity-dependent biotin identification (BioID) is a powerful tool to interrogate protein-protein interaction networks. BioID has been successful in dissecting the networks involved in centriole duplication and elongation.^11,27–29^ Pelletier and colleagues mapped the molecular vicinity of centriole, centriolar satellite, and ciliary transition zone proteins, thereby identifying >1,700 unique proteins.^30^ Although effective, BioID and other proximity-dependent labeling methods have certain limitations; for instance, interactions occurring at centrosomes or elsewhere in cells are indistinguishable, while tagging and overexpression of target proteins could impact not only their normal interactions but centrosome organization itself.

These proteomic studies of centrosomes have revealed many shared components but also unveiled differences, pointing to possible cell-type- and tissue-specific functions of centrosome proteins. For instance, the centriolar protein proteome of centriole 1 (POC1) has two human paralogs, POC1A and POC1B. Whereas inherited mutations in *POC1A* cause a rare type of primordial dwarfism,^31^ individuals with mutations in *POC1B* develop cone-rod dystrophy, an eye disorder associated with vision loss.^32,33^ Furthermore, we recently reported that the ubiquitously expressed PCM scaffolding protein CDK5RAP2 is required for mitotic spindle formation in erythroblasts but not in fibroblasts, implying differences in PCM function between cell types.^34^ Finally, immunoprecipitation-based profiling of centro-somal protein complexes revealed intriguing differences between centrosomal protein-protein interaction networks of neural stem cells and differentiated neurons.^35^ Considering these recent discoveries and the limitations of current approaches, there is a clear need for alternative methods that allow centrosome enrichment from a range of cell types for proteome profiling.

To this end, we have developed a single-step affinity purification method called CAPture that enables centrosome enrichment from a variety of cell types including transformed and primary cells. MS-based analysis of affinity-purified centrosomes revealed a substantial overlap with previously published centrosome proteomes while identifying over 50 additional candidate proteins. We demonstrate that CAPture combined with MS is highly sensitive and specific to centrosomes and, as such, represents a powerful tool to dissect hierarchical relationships in centrosome assembly. In addition, our results uncover discernible differences in centrosome composition between different cell types, substantiating the existence of tissue- and/or cell-type-specific components. The ability of CAPture to identify these compositional differences in a technically straightforward way will facilitate progress toward understanding the physiological and pathological roles of centrosomes.

## Results

### CAPture, an affinity-based method, can isolate intact centrosomes from cell lysates

**Figure 1:**
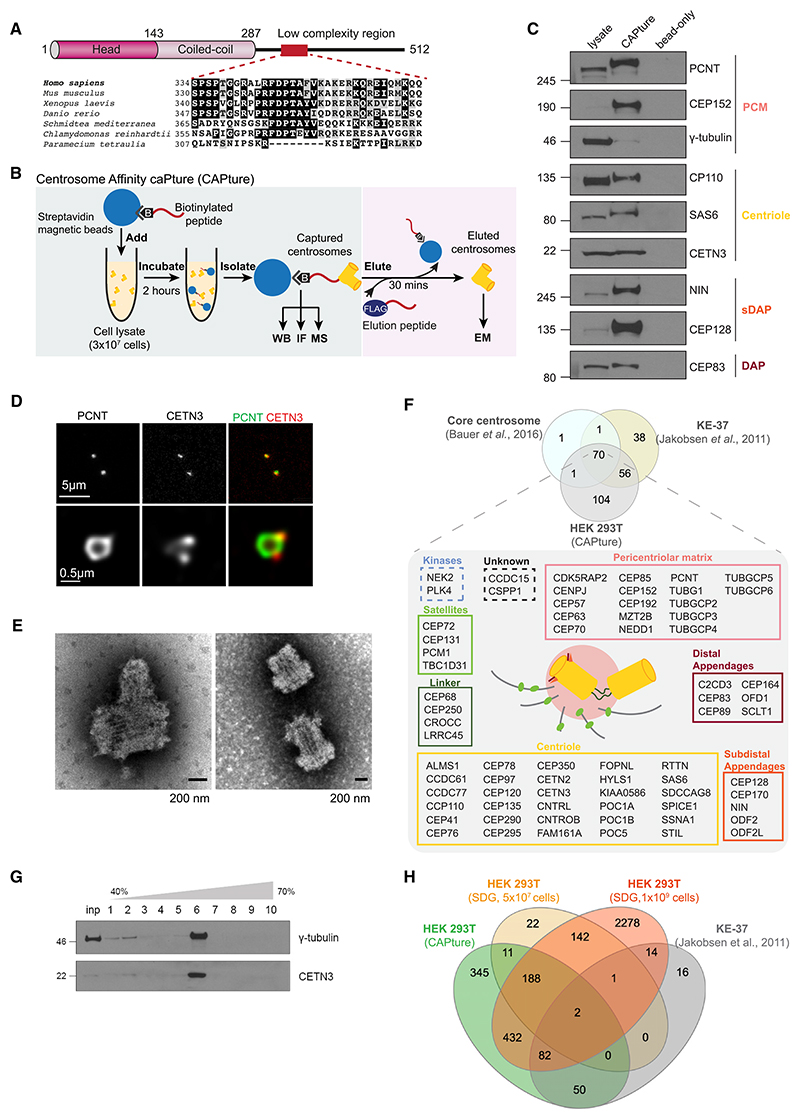
Intact centrosomes are isolated from HEK293T cells with centrosome affinity capture (CAPture) (A) CCDC61 contains a conserved low-complexity region (CCDC61-LCR). (B) CAPture utilizes a biotinylated peptide derived from CCDC61-LCR (called CAPture peptide from hereon) to isolate centrosomes from sonicated whole-cell lysates. Isolated centrosomes were subjected to western blotting (WB), mass spectrometry (MS), immunofluorescence (IF), or eluted from beads for electron microscopy (EM). (C) Western blots show enrichment of known centrosome components by CAPture from HEK293T cells. Components of distinct centrosome substructures such as PCM, centriole, or appendages are represented. Lysate: whole-cell lysate. CAPture lane: proteins bound to CAPture peptide-streptavidin bead complexes. Bead-only lane: proteins associated with streptavidin beads. Same nomenclature used throughout all figures. (D) Bead-bound centrosome imaged by stimulated emission depletion (STED) microscopy. Centrosomes were co-stained with pericentrin (PCNT) and centrin-3 (CETN3). (E) Negative stain electron micrograph of centrosomes obtained by CAPture from FreeStyle 293-F cells. (F) Mass spectrometry (MS) analysis of CAPture from HEK293T cells. Venn diagram shows comparison of centrosome proteome obtained by CAPture-MS in HEK293T cells with the core^25^ and KE-37 centrosome proteomes both obtained by sucrose density gradient (SDG) centrifugation.^24^ See text for proteome inclusion criteria. (G) Western blots show fractions following consecutive SDG centrifugation. Input was collected after first centrifugation (50% sucrose cushion). Centrosomes are expected to sediment between 50% and 70% on discontinuous sucrose gradient (% sucrose is indicated above blots). Fraction 6 contains highest levels of the centrosomal proteins, centrin (CETN3) and γ-tubulin. (H) Venn diagram shows comparison of proteomes obtained by CAPture from HEK293T cells (not corrected for bead-only binders) or SDG from 5 × 10^7^ or 10^9^ HEK293T cells with the published KE-37 centrosome proteome derived by SDG.^24^ n = 1 for each sample. For further details see Figures S1B–S1D.

Our previous structural work revealed a low-complexity region (LCR) of ~240 residues in the centrosome component, coiled-coil-domain-containing protein 61 (CCDC61, also known as variable flagellar number 3)^36^ (Figure 1A). We hypothesized that a central conserved stretch of this domain could mediate important interactions of CCDC61 and generated synthetic biotinylated peptides to affinity-purify binding partners. Pull-down from HEK293T cell lysate with a 33-residue-long peptide (CCDC61-LCR) resulted in enrichment of several centrosomes proteins, whereas no centrosomal proteins were purified by streptavidin beads (bead-only) (Figures 1B and 1C). Since the peptide enriched proteins from several centrosome substructures (i.e., centriole, PCM) we suspected that it interacts with centrosomes rather than soluble fractions of CCDC61-binding partners. Indeed, stimulated emission depletion (STED) and negative stain electron microscopy (EM) confirmed association of peptide-bound streptavidin beads with centrosomes containing single or duplicating centrioles (Figures 1D and 1E). Given the ability of the peptide to affinity-purify centrosomes, we call this method centrosome affinity capture (CAPture) and refer to the 33-residue-long CCDC61-LCR peptide as CAP-ture peptide.

We next prepared 3 biological replicates of CAPture from 3 ×10^7^ HEK293T cells along with 2 negative control samples (bead-only pull-down) for MS (Table S1). In total 846 proteins were detected in the bead-only samples including 5 centrosomal proteins (CCDC61, ODF2, CEP128, centrin-2 [CETN2], and CEP170). Only CCDC61 was identified in both samples with 1 and 2 unique peptides, respectively. The presence of these proteins in bead-only samples is probably due to weak non-specific hydrophobic binding to the beads because they were detected with much fewer unique peptides in bead-only than CAPture-MS samples (Table S1). Following removal of bead-only-bound proteins (except for the aforementioned five), CAPture-MS identified 231 proteins by at least 1 unique peptide in 3 biological replicates; these included 71 of 73 known core centrosome components,^25^ and 126 proteins of the 165 proteins previously found in the centrosome proteome of KE-37 lymphoblasts^24^ (Figure 1F). Furthermore, 151 proteins were shared between CAPture-MS and the centrosome-cilia proximal interacting network obtained by BioID from HEK293T cells^30^ (Figure S1A), indicating that CAPture-MS yields reproducible centrosome proteomes from 20 to 30 million HEK293T cells. Although centriolar satellites are highly enriched in centrosomal proteins,^37,38^ the core centriolar satellite component, PCM1, was not among the top 50 hits in CAPture-MS of HEK293T cells, suggesting that CAPture does not isolate satellites.

### CAPture-MS outperforms sucrose density gradient (SDG)-dependent centrosome isolation both in sensitivity and in specificity

We next compared performance of CAPture-MS with that of SDG-dependent centrosome enrichment. Centrosomes were isolated from 5 × 10^7^ HEK293T cells and processed for MS; for CAPture, samples were digested on beads, whereas for SDG, in-solution digest was performed on fractions most enriched for centrosomes as judged by immunoblotting for γ-tubulin and CETN3 (fraction 6, Figure 1G). A total of 1,110 proteins were identified by CAPture-MS of which 134 proteins were shared with the published centrosome proteome derived by SDG and quantitative MS from the KE-37 human T cell leukemia cell line.^24^ MS of fraction 6 from SDG of HEK293T found only 3 proteins common with the KE-37 dataset (Figure 1H). Using 10^9^ HEK293T cells for SDG improved the overlap by detecting 99 proteins from the KE-37 dataset. However, in total 3,139 proteins were identified, showing poor specificity relative to CAPture-MS (Figures S1B–S1D; Table S2), especially because after removal of bead-only binders from the CAPture-MS data, only 539 proteins remain of which 134 (~25%) are shared with the KE-37 centrosome proteome. CAPture-MS also yielded much greater peptide numbers of centrosome components than SDG (Figures S1B and S1C). Thus, CAPture provides increased sensitivity and specificity for MS-based detection of centrosome composition.

### Efficiency of CAPture is greatly impaired from cells depleted of centrioles

**Figure 2:**
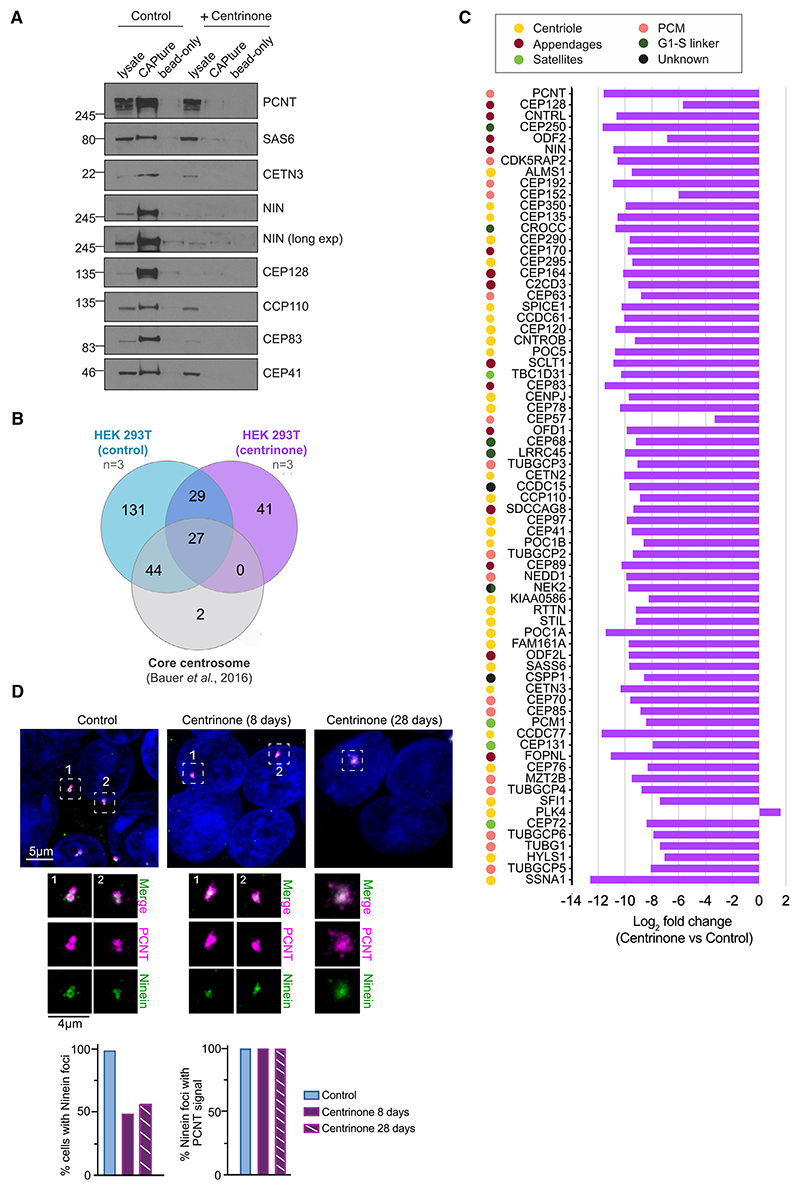
CAPture isolates intact centrosomes and not soluble centrosomal protein complexes (A) Centrosomal protein retrieval by CAPture is reduced from centrinone-treated cells. Western blots show levels of select centrosome components in lysates, CAPture peptide-bound and bead-only fractions. A longer exposure (long exp) image for Ninein is included. (B) Venn diagram shows comparison of centrosomes proteomes obtained by CAPture-MS from control and centrinone-treated HEK293T cells with the published core centrosome proteome.^25^ See text for proteome inclusion criteria. Note that control datasets were used to generate data in Figure 1F. (C) Bar chart depicts log_2_-fold change in core centrosomal proteins^25^ between CAPture-MS of centrinone-treated vs. control HEK293T cells obtained by LFQ analysis (Table S1). Note that all values shown are statistically significant (p_adj_ < 0.05). Proteins in chart are ordered as per their protein score in control (PCNT being highest, SSNA1 lowest) and color-coded according to the centrosomal sub-structure they associate with. (D) Confocal micrographs of control HEK293T cells or following centrinone treatment for 8 or 28 days. Ninein (green) co-localized with PCNT (magenta) in cytoplasmic foci. Bar graphs below show percentage of cells with Ninein foci (left) and percentage of Ninein foci positive for PCNT staining (right). For Ninein and Ninein/PCNT co-localization >230 and >40 cells were scored per condition, respectively.

Our results so far suggest that CAPture can isolate centrosomes, but it is unclear if it also interacts with cytoplasmic pools of centrosomal proteins. To address this, we compared performance of CAPture from HEK293T cells with and without centrioles. After 8 days of treatment with centrinone, an inhibitor of PLK4 that prevents centriole assembly,^39^ both parental centriole (CEP152) and procentriole (SAS-6) markers were largely absent (Figure S2A). Although western blots of CAPture from centrinone-treated cells showed minimal enrichment of centrosomal proteins over bead-only levels (Figure 2A), CAPture-MS still identified a few known centrosome components (Table S1). In control proteomes (derived from n = 3 CAPture and n = 2 bead-only), 71 of 73 core centrosomal proteins^25^ were detected with at least one peptide in all 3 replicates. Note that same control samples were used for Figure 1F. By contrast, in centrinone-treated proteomes (n = 3 CAPture and n = 2 bead-only), only 27 core centrosomal proteins were found even when including proteins present in just 2 replicates (Figure 2B). Moreover, label-free quantitative (LFQ) analysis confirmed significant depletion of all core centrosomal proteins in the centrinone samples (Figure 2C; Table S1) with PLK4 being the only exception. Therefore, centrosomal proteins are either undetectable and/or markedly reduced in CAPture-MS datasets of centrinone-treated cells, arguing against strong interactions between the CAPture peptide and its binding partner(s) outside of the centrosome context.

What could be the source of the few centrosomal proteins still detectable in CAPture-MS of centrinone-treated cells? Although residual centrioles may persist in ~1%–2% of cells after 8 days in centrinone, even after 28 days a small amount of CEP128 was visible on immunoblots of CAPture despite EM confirming centriole loss in CAPture samples (Figures 2A, S2B, and S2C). Instead of residual centrioles, the occasional centrosomal protein seen in CAPture-MS may originate from the pull-down of acentriolar protein assemblies, structures previously observed in centrinone-treated cells.^40^ Indeed, both 8- and 28-day centrinone treatment triggered formation of compact cytoplasmic foci in interphase HEK293T cells, which contained varying amounts of pericentrin (PCNT), CEP128, Ninein, and CEP192 (Figures 2C, 2D, S2D, and S2E). By blocking auto-phosphorylation-dependent degradation of PLK4,^41,42^ centrinone is known to elevate PLK4 protein levels which could trigger accumulation of the kinase in these acentriolar assemblies, probably explaining its presence in the MS data. Nonetheless, our results imply that while the CAPture peptide may bind some centrinone-induced acentriolar assemblies, its performance is vastly superior in centrosome-containing cells, indicative of a clear preference for interactor(s) located within an intact centrosome.

### The centrosomal protein Ninein is vital for CAPture

**Figure 3:**
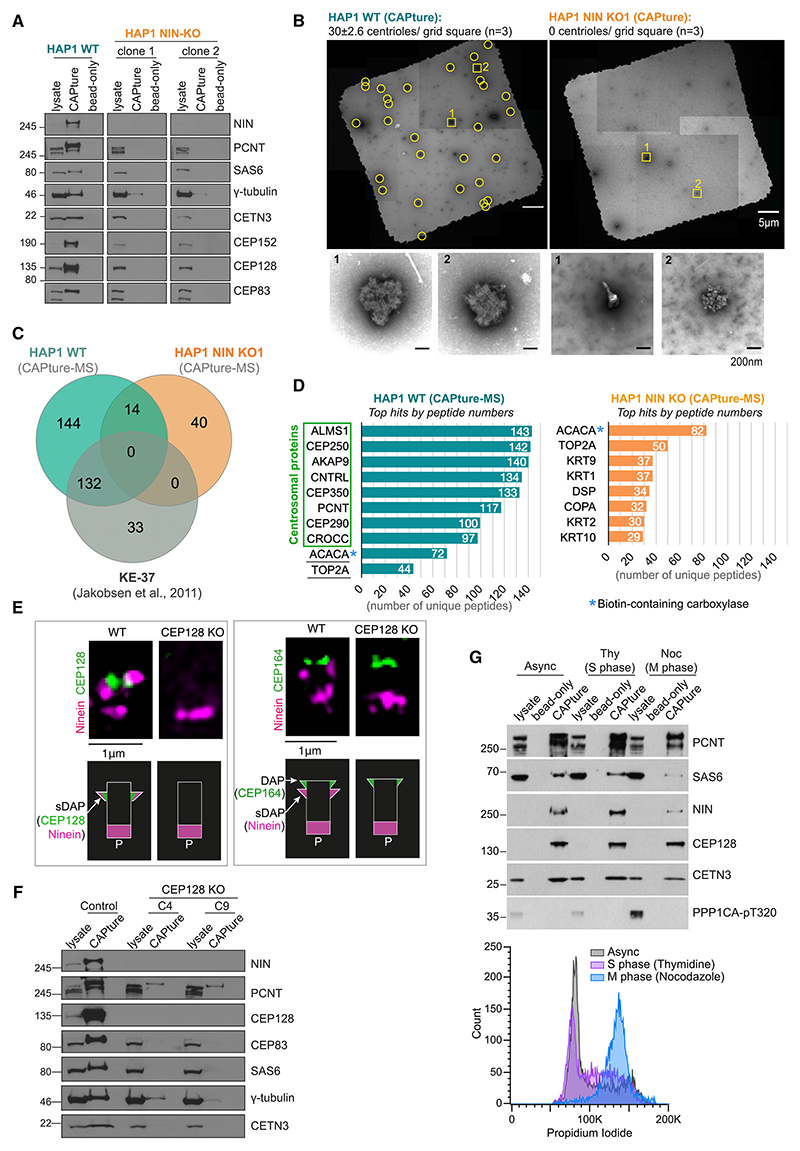
Centrosome isolation by CAPture requires Ninein (A) Loss of centrosomal protein retrieval by CAPture from Ninein knockout (KO) HAP1 cells (B4 and B12 clones).Western blots show select centrosome components in lysates, CAPture-bound, and bead-only fractions. (B) Negative stain electron micrographs showing the grid squares of WT and NIN-KO centrosome samples obtained by CAPture. Circles indicate centrioles. Magnified views of yellow squares in the micrographs correspond to centrioles in WT and examples of electron-dense aggregates in NIN-KO. (C) Venn diagram shows comparison of centrosome proteomes obtained by CAPture from HAP1 WT and HAP1 NIN-KO cells (n = 3 per genotype, inclusion criterium is ≥1 unique peptide in all replicates) with the published KE-37 centrosome proteome.^24^ (D) Bar charts depict proteins detected with greatest unique peptide numbers (average of 3 experiments) for each sample. To facilitate comparison, for CAPture-MS of HAP1 WT, ACACA, and TOP2A (underlined) are included despite ranking 15th and 28th, respectively. (E) Structured illumination microscopy (SIM) of WT and CEP128 KO centrosomes in intact HEK293T cells. Ninein is retained only at the proximal (P) end of centrioles in CEP128 KO cells as shown by co-staining of centrosomes with Ninein (magenta) and CEP128 (green) on top or Ninein (magenta) with the DAP marker CEP164 (green) on bottom. Schematics to aid orientation are shown below. (F) CAPture efficiency is impaired in CEP128 knockout (KO) HEK293T cells. Western blots show levels of various centrosome components in lysates and CAPture-bound fractions from WT and CEP128-KO clones. (G) CAPture performs similarly from asynchronous (async) HEK293T cells or those arrested in S-phase (two rounds of thymidine treatment) or M-phase (thymidine followed by monastrol treatment). Western blots show levels of various centrosome components in cell lysates and in CAPture-bound fractions. Corresponding flow cytometry profiles are shown below.

CCDC61 has been reported to interact with the sDAP protein CEP170, which in turn is a binding partner of Ninein.^43,44^ Given the prevalence of Ninein in both acentrosomal assemblies and CAPture-MS of centrinone-treated cells (Figures 2C and 2D), we tested its contribution to the CAPture method by generating Ninein knockouts (NIN-KOs) in HAP1, a near-haploid chronic myelogenous leukemia cell line. Both NIN-KO clones contained frameshift mutations and lacked an obvious band by western blotting or a centrosomal signal by immunofluorescence (IF) while proliferating normally (Figures 3A and S3A–S3C). Western blots and EM analysis of CAPture samples from NIN-KO HAP1 cells showed a lack of enrichment of centrosomal proteins and absence of centrioles, respectively (Figures 3A and 3B). CAPture-MS performed from WT and NIN-KO cells (n = 3 CAP-ture with n = 1 bead-only control per genotype) revealed 289 proteins in WT that were identified by at least one peptide in each of 3 replicates while absent from the bead-only sample; these include all 73 core centrosomal proteins,^25^ and 132 of the 165 proteins identified from KE-37 cells^24^ (Figure 3C; Table S3). The same selection criterium applied to NIN-KO data yielded 54 proteins but no known centrosome components (Figures 3C and 3D; Table S3). Note that despite being present in all 3 replicates, CCDC61 was excluded because the 4 peptides identified all mapped to the CAPture peptide and so likely originate from digestion of bead-peptide complexes. Only 3 normally highly abundant centrosomal proteins were detected in NIN-KO: ODF2 in two replicates with 1 and 3 unique peptides whereas A-kinase anchor protein 9 (AKAP9) and CROCC in only one replicate and with one peptide. Of the 54 proteins identified in NIN-KO, 32 were seen in bead-only samples of WT HAP1 or HEK293T cells, whereas 15 were shared with CAPture-MS of HEK293T. The latter likely represent genuine non-centrosomal interactors of CAPture peptide-bead complexes and include the COPI coatomer complex and CRBN, levels of which were comparable between control and centrinone-treated cells in LFQ analysis (Table S1). Therefore, all subsequent centrosome proteomes were filtered against these 54 proteins, unless otherwise stated. Importantly, proteins identified by the highest peptide numbers were identical between bead-only samples of WT HAP1 and NIN-KO cells and corresponded to endogenous biotin-containing proteins (e.g., ACACA and PC) that bind streptavidin beads (Figure S3D).

Ninein and CEP170 exist in two distinct centrosomal pools; their sDAP-associated pool depends on CEP128, a protein essential for sDAP formation, whereas their pool at the proximal ends of parental centrioles is independent of CEP128.^45–47^ To distinguish between contribution of these two Ninein pools to CAPture, we deleted CEP128 in HEK293T cells. Two CEP128-KO clones contained identical biallelic frameshift mutations (Figure S3E) with CEP128 being absent from centrosomes (Figure 3E) and cell lysates (Figure 3F). As expected, Ninein was present only at the the proximal ends of centrioles in these cells (Figure 3E). When compared with control, CAPture from CEP128-KO cells resulted in much lower yields of centrosomal proteins (Figure 3F). In some experiments the CEP128 antibodies reacted weakly with a band on immunoblots of CAPture from CEP128-KO; we were unable to confirm specificity of this band, but the frameshift mutations preclude presence of full-length CEP128 (Figure S3F). Nonetheless, enrichment of PCNT suggests that despite absence of CEP128 CAPture can still isolate some centrosomes.

Altogether, our data indicate that Ninein or a Ninein-dependent complex provides the specificity for CAPture-dependent centrosome isolation. Dependency of CAPture on CEP128 high-lights a crucial contribution by sDAP-associated Ninein. However, sDAPs undergo dynamic changes during the cell cycle; through G1- and S-phases only mother centrioles possess sDAPs with daughter centrioles acquiring these structures in G2. sDAPs are then remodeled in mitosis^48^ resulting in CEP170 and Ninein being detectable only at the PCM.^49,50^ Despite these cell cycle-dependent changes, CAPture provided effective centrosome enrichment from both S- and M-phase-synchronized HEK293T cells (Figure 3G). While caution must be exercised due to inherent limitation of synchronization protocols, the ability of CAPture to isolate centrosomes even from mitotic cells where Ninein is exclusively at the PCM^49^ suggests that the requirement for Ninein is independent of the exact sub-centrosomal localization of the bulk of its pool. Furthermore, centrosomal levels of many proteins are subject to cell-cycle-dependent control, likely contributing to differences in CAPture enrichment profiles across the cell cycle.

### CAPture-MS enables comparison of centrosomes proteomes between cell types

**Figure 4:**
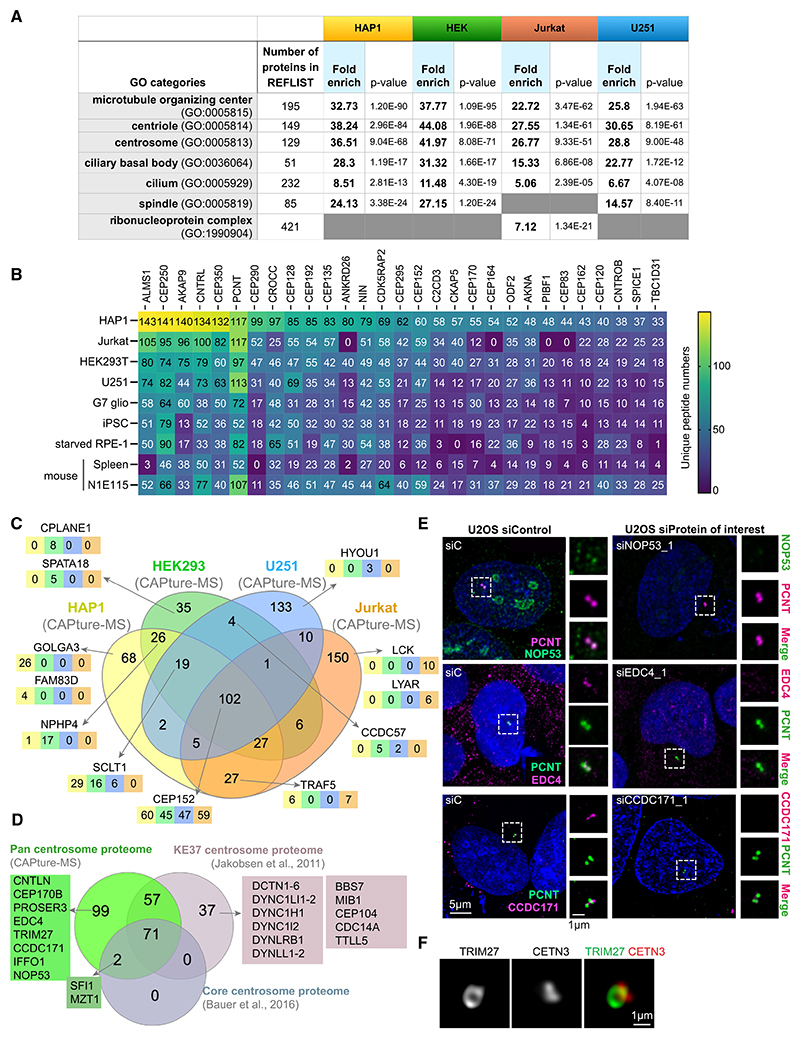
CAPture-MS reveals cell-type-specific differences in centrosome proteomes (A) Gene ontology (GO) based enrichment analyses of centrosome proteomes obtained by CAPture-MS from cell lines as indicated. PANTHER over-representation test was performed on GO-slim Cellular Components annotation dataset. Fold enrichment and p values (Fisher’s exact Bonferroni corrected) are shown. Criteria for inclusion in datasets: proteins detected by at least one peptide in all CAPture-MS repeats (n = 3 for HAP1 or HEK293T cells, n = 2 for Jurkat and U251), while being absent from cell-type-matched bead-only binders and from non-centrosomal binders of CAPture peptide (based on HAP1 NIN-KO). (B) Heatmap depicts the 25 proteins identified with highest average unique peptide numbers in HAP1 cells. Average number of unique peptides is shown for HAP1 (n = 3), HEK293T (n = 3), Jurkat (n = 2), U251 (n = 2) and mouse spleen (n = 2). For serum-starved RPE-1, G7 glioblastoma cells, iPSCs, and N1E-115 mouse neuroblastoma cells data correspond to single experimental repeat. (C) Venn diagram depicts comparison of centrosome proteomes obtained by CAPture from HEK293T (green), HAP1 (yellow), U251 (blue), and Jurkat (orange) cells. Examples for proteins with differential distribution are shown with the average number of unique peptides identified in each cell line (color-coded); unique peptide numbers are set at 0 for proteins that are not detected in at least 2 repeats of a particular cell line. Datasets and rules for protein inclusion are same as in (A). (D) Venn diagram comparing the pan-centrosome proteome (proteins present in centrosome proteomes of at least two cell lines according to criteria in A) to published KE-37 centrosome proteome.^24^ Examples for proteins unique to each sample are shown. (E) Validation of candidate proteins from pan-centrosome proteome. Confocal micrographs show NOP53 and CCDC171 staining in mock-depleted (siC) U2OS cells or in cells depleted of NOP53 (siNOP53) or CCDC171 (siCCDC171). Candidates are co-stained with PCNT, a PCM marker. Further staining and analyses are in Figure S4. (F) Deconvolved confocal micrograph of a centrosome isolated from HEK293T cells by CAPture, spun onto coverslips and stained with antibodies against TRIM27 (green) and CETN3 (red).

We have demonstrated that CAPture isolates intact centrosomes from HAP1 and HEK293T cell lysates in a manner dependent on sDAPs and Ninein. As Ninein is widely expressed in mammalian tissues,^51^ we suspected that CAPture could be useful to study centrosome proteomes across multiple cell types. To this end, we performed 2 biological replicates of CAPture-MS from 3 × 10^7^ Jurkat (T cell leukemia) and U251 (established glioblastoma [GBM] cell line) and compared these proteomes (Table S4) with those of HAP1 and HEK293T centrosomes (Table S3). In both U251 and Jurkat, CAPture-MS identified over 200 proteins detected by at least one unique peptide in two replicates. Once filtered against bead-only samples and non-centrosomal binders of the CAPture peptide from HAP1 NIN-KO cells, centrosome proteomes were subjected to gene ontology (GO) enrichment analysis. In all cell lines the GO terms microtubule-organizing center, centriole, and centrosome were highly enriched (25- to 45-fold) with adjusted p values of <10^−48^ (Figure 4A), underscoring specificity of the method. When proteins were ranked according to unique peptide numbers in CAPture-MS of HAP1, the top ranked candidates were also abundant in CAPture-MS of Jurkat, U251, and HEK293T cells with some notable exceptions (Figure 4B). For instance, ANKRD26, identified with 13–80 unique peptides in HAP1, HEK293T, and U251 cells, was absent from Jurkat cells.

Along with components unique to cell lines, CAPture-MS un-covered 102 proteins shared by all four centrosome proteomes (Figure 4C). 229 proteins were common between at least two cell lines, and we named this dataset the pan-centrosome proteome (Figure 4D). The latter contains all 73 core centrosomal proteins^25^ and an additional 99 proteins that were not identified in the KE-37 centrosome proteome.^24^ These include several recently described centrosome components but also additional candidates (Figure 4D). Using confocal microscopy (Figures 4E and S4) combined with RNA-interference-mediated depletion, we confirmed centrosomal association of enhancer of mRNA decapping 4 (EDC4), CCDC171 and the nucleolar protein NOP53. Tripartite-motif-containing 27 (TRIM27) was detected by STED microscopy of isolated centrosomes (Figure 4F). Along with a few known centrosomal proteins (i.e., CROCC, ODF2, and HERC2), NOP53 was present in some negative control samples (e.g., bead-only of Jurkat and U251 but not HAP1 or HEK293T cells), suggesting that it may stick to streptavidin beads in certain cell types. However, LFQ analysis of CAPture-MS from control and centrinone-treated HEK293T cells revealed enrichment of NOP53 in the former (5 unique peptides, log_2_fold change: 3.04, p_adj_ = 0.0043, Table S1), consistent with centrosomal localization of NOP53 (Figure 4E). Therefore, in some cases quantitative methods are needed to distinguish between genuine centrosomal proteins and background.

Despite extensive overlap with the published KE-37 centrosome proteome,^24^ dynein complexes and their associated proteins identified from KE-37 cells could not be observed by CAPture-MS (Figure 4D). To exclude that absence of dynein complexes is due to differences in the underlying experimental procedures, we combined CAPture-MS of Jurkat cells with nocodazole and cytochalasin treatments historically used in SDG-based centrosome isolation to depolymerize microtubules and actin. However, these changes did not facilitate detection of dynein complexes (Table S4). While some kinesins were detectable by CAPture-MS (KIF2A, KIF22, and KIF23), it is possible that the salt concentration (300 mM NaCl) of CAPture buffer removes dynein complexes from centrosomes.

In summary, we demonstrated that CAPture performs comparably across four different cell lines, yielding high-resolution cell-type-specific centrosome proteomes.

### Proteomic analysis of centrosomes from primary cells and tissues

The cell lines tested with CAPture-MS so far were all fast-growing cancer cell lines. To assess whether the method was also successful in primary and untransformed cells, we selected a small panel of cell lines that included Fs13b induced pluripotent stem cells (iPSCs), a GBM-derived neural stem cell line G7 and untransformed human telomerase reverse transcriptase (hTERT)-immortalized retinal pigment epithelial (RPE)-1 cells. In quiescence, many cell types assemble a primary cilium by converting the appendage-bearing mother centriole into a basal body, which subsequently nucleates the ciliary axoneme. Cilia were detectable both in G7 and iPSCs (20% and 4% ciliated cells, respectively), but they were even more prevalent in serum-starved RPE-1 cells (>80%). Because CAPture relies on Ninein and sDAPs in centrosomes, and these components are retained in the basal body of primary cilia,^46,52^ we asked whether CAPture could isolate basal bodies from serum-starved RPE-1 cells. Using cryoelectron tomography (cryo-ET), we observed striated filaments reminiscent of ciliary rootlets in a centriole pair, which are a feature of basal bodies (Figure S5A). As reported previously,^53,54^ ice-embedded basal bodies were compressed and therefore the centriolar triplet microtubule array showed an oval arrangement (magenta in Figure S5A). These results are consistent with CAPture’s ability to isolate basal bodies, albeit its efficiency remains unclear.

CAPture-MS from G7, iPSCs, and serum-starved RPE-1 cells (3 × 10^7^, ~1–2 × 10^7^, and 4 × 10^7^ cells, respectively) yielded excellent overlap with previous centrosome proteomes (Figures 4B and S5B–S5D; Table S5). All 3 cell lines showed at least 12-fold enrichment for GO terms of microtubule-organizing center, centrioles, and centrosome with adjusted p values of <10^-29^ (Figure S5C). Intriguingly, CCP110 and CEP97, two ciliogenesis-blocking factors,^55^ were lacking from CAPture-MS of RPE-1 cells, but further repeats will be necessary to substantiate these findings (Figure S5D).

To address whether CAPture-MS could work on complex primary samples, we trialed two organs from mice: liver and spleen. For liver, only a handful of centrosomal proteins were detected with several being also present in the bead-only sample. Interestingly, CAPture also failed in the human hepatocyte cell line, HepG2, consistent with a potential tissue-specific cause. By contrast, performance of CAPture seemed promising in spleen lysed by rotor-stator homogenization. Including proteins identified by at least one peptide in two repeats, CAPture-MS in adult spleen detected 55 of 73 core and 87 of 165 KE-37 centrosomal proteins^24^ (Figure S5D). Accordingly, highly significant enrichment was seen for centrosome-related GO terms (Figure S5C).

The top hits from HAP1 centrosomes were also found in iPSC and spleen, albeit overall peptide counts tended to be lower in iPSC and spleen perhaps due to difficult-to-control cell numbers and higher background from the tissue (Figure 4B). Consistent with its roles as a blood filter and lymphoid organ, mouse spleen comprises a large number of cell types ranging from blood cells (i.e., granulocytes and erythrocytes), endothelial and stromal cells to lymphocytes, plasma, and even smooth muscle cells. Most of these cell types lack cilia, and thus, it is interesting that spleen centrosomes showed low peptide counts for ciliogenesis-linked proteins such as ALMS1 or DAP components that form the outer parts of appendages (CEP164, SCLT1, and ANKRD26; Figure S5E). Lower detection was not due to species differences because the same proteins were abundant in CAPture-MS of N1E-115, a mouse neuroblastoma cell line (Figures 4B and S5E). In summary, we have demonstrated that CAPture-MS can yield valuable centrosome proteomes from untransformed cells and also from complex cell mixtures present in primary tissues.

### Identification of cell-type-specific differences in centrosome proteomes

Despite the extensive overlap between centrosome proteomes of different cell lines (Figures 4C and 4D), we also noticed reproducible differences. For instance, GOLGA3 and CPLANE1 were found in CAPture-MS of only HAP1 and HEK293T cells, respectively, whereas Jurkat centrosomes uniquely lacked several DAP components including OFD1 and CEP83 (Figure 4B).

**Figure 5:**
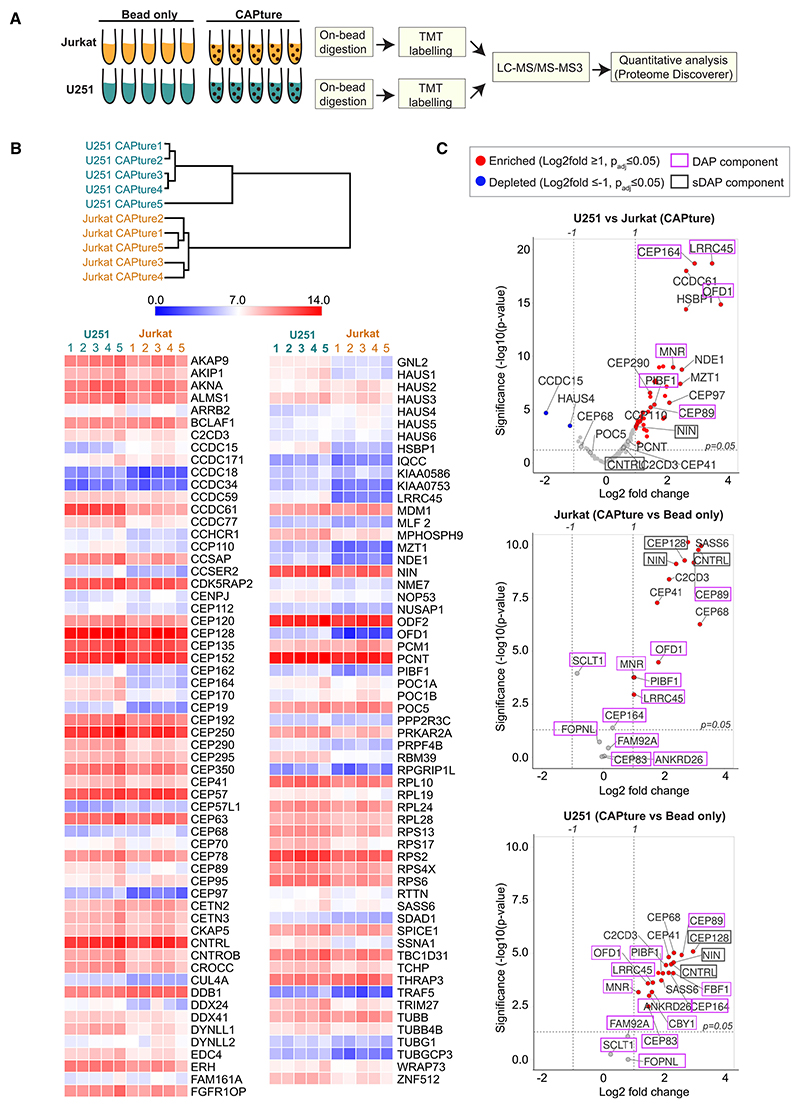
Tandem mass tag (TMT)-based quantitative proteomic profiling reveals marked differences in protein enrichment between centrosomes obtained by CAPture from U251 and Jurkat cells (A) Experimental outline of two 11-plex TMT experiments. (B) Hierarchical clustering of the CAPture samples is shown on top. Heatmap below depicts normalized TMT signal intensities of the 119 proteins quantified in U251 and Jurkat centrosomes. Unlike data in Table S6, this dataset was filtered for non-centrosomal binders of the CAPture peptide (based on HAP1 NIN-KO samples). See text for inclusion criteria. (C) Volcano plots depict log_2_-fold change in normalized TMT signal intensities vs. −log_10_(adjusted p value) with threshold for significance set at p = 0.05. Top plot shows 119 proteins quantified in Jurkat and U251 centrosomes. Select centrosomal proteins including all quantified DAP components (purple frame) are labeled. Middle and bottom plots depict quantification of DAP components and select centrosomal proteins in CAPture-MS vs. bead-only MS of Jurkat and U251 cells, respectively.

As unique peptide number and coverage in MS are only an approximation of protein abundance, quantitative methods are needed to compare centrosome proteomes between different cell types. To this end, we performed two sets of 11-plex isobaric tandem mass tag (TMT) labeling experiments on CAPture and bead-only samples of U251 and Jurkat cells (Figure 5A). This experimental design also provided quantitative data on the performance of CAPture peptide over bead-only within each cell line. In total, 10,488 peptides and 1,447 proteins were identified in U251 cells, and 4,823 peptides and 700 proteins were detected in Jurkat cells. Following removal of non-centrosomal binders of the CAPture peptide (based on NIN-KO HAP1), 136 proteins in U251 and 176 in Jurkat were significantly and at least 2-fold enriched (p_adj_ < 0.05 following normalization) in CAPture vs. bead-only samples (Table S6), including several candidate proteins from Figures 4D–4F (Figure S6A). Statistically significant enrichment over bead-only samples was seen for 149 and 122 components identified previously in the Jurkat and U251 shotgun centrosome proteomes, respectively. 1,088 peptides and 125 proteins (119 following removal of non-centrosomal binders based on NIN-KO HAP1) were quantified between the U251 and Jurkat CAPture sets (Figure 5B; Table S6). Cross set comparisons could only be performed on proteins detected in both cell lines, and only when they showed significant enrichment over cell-line-matched bead-only samples. Hierarchical clustering analysis based on TMT signal intensities of centrosomal proteins showed samples from same cell lines being clustered together (Figure 5B). 44 proteins exhibited a statistically significant fold change of R2 between centrosomes of the two cell lines with 20 displaying a fold change of 3 or over (Figure 5C, top plot). The latter included OFD1, LRCC45, and CEP164 that were 6-to 10-fold enriched in U251 vs. Jurkat centrosomes.

**Figure 6:**
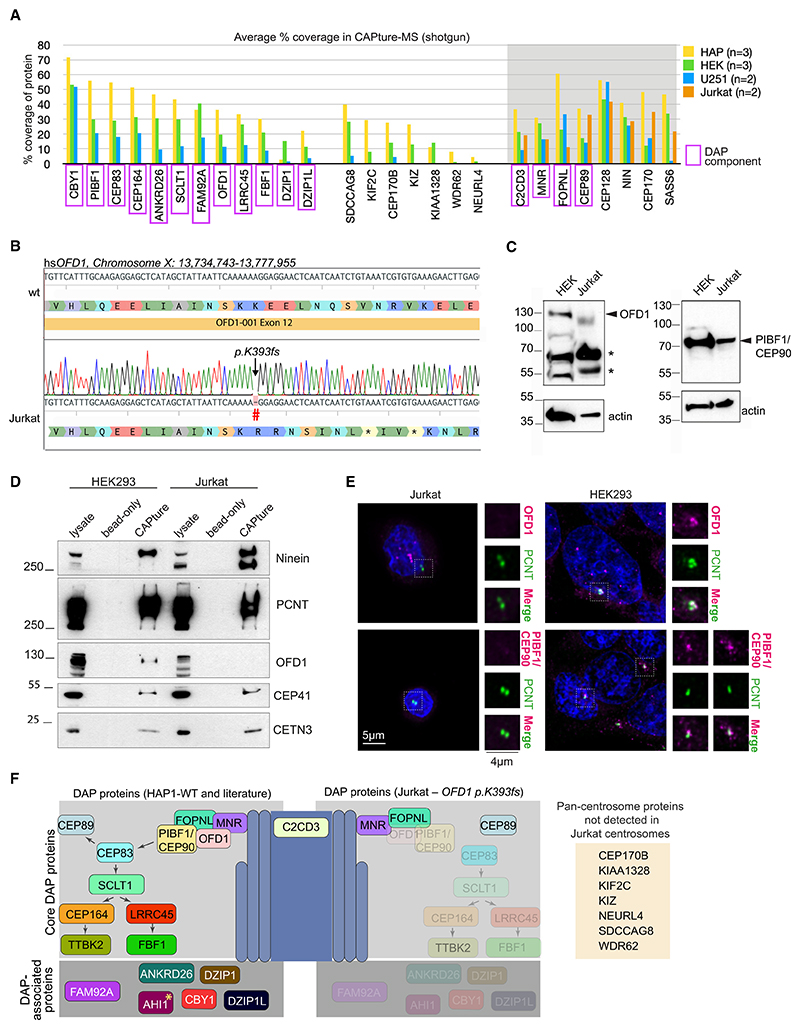
The DAP protein network is compromised in Jurkat cells by a naturally occurring frameshift mutation in *OFD1* (A) Chart depicts proteins present in the pan but absent from the Jurkat centrosome proteome. Proteins are considered missing if they are absent from at least two repeats of Jurkat CAPture-MS (shotgun) and show no enrichment over bead-only samples in Jurkat CAPture-TMT. Note absence of most known DAP proteins (purple frame) from Jurkat centrosomes along with several proteins with no previous link to DAPs. Shaded area on chart depicts examples for proteins shared between cell lines; MNR, FOPNL, CEP89, are DAP components, CEP128, CEP170, and NIN are sDAP components and SAS-6 associates with procentrioles. (B) Sequencing of Jurkat *OFD1* genomic locus reveals frameshifting point mutation in exon 12 (transcript ID: ENST00000340096.6). (C) Western blots of OFD1 (left) and PIBF1/CEP90 (right) in HEK293T and Jurkat cell lysates. Actin serves as loading control. Note lack of clear OFD1 band at expected size in Jurkat cells. Asterisks mark bands that may be non-specific, or at least do not appear in isolated centrosomes based on (D). (D) OFD1 is enriched in centrosomes of U251 but not Jurkat cells based on CAPture-WB. Immunoblots show levels of OFD1 and other centrosome components in lysates and CAPture peptide-bound fractions. Note that despite antibody recognizing multiple bands in both lysates, a single band is visible in HEK293T centrosomes. (E) Confocal micrographs depict OFD1 (magenta) and PIBF1/CEP90 (magenta) localization in Jurkat and HEK293T cells. Cells were co-stained with PCNT (green), a PCM marker. Higher magnification images of centrosomes are shown on the right. Note absence of OFD1 and PIBF1 signal in Jurkat centrosomes. (F) Cartoon on left depicts network of known core DAP components and DAP-associated proteins along with their recruitment hierarchy where known (left). Except for AHI1, all reported DAP components have been detected in CAPture-MS from HAP1 cells (A; Table S3). Cartoon on right shows presence/absence of particular DAP components in Jurkat centrosomes (Table S4). Box on right lists proteins from the pan-centrosome proteome that are absent from Jurkat centrosomes but not known to associate with DAPs.

Unlike in U251, there was a lack of enrichment of DAP components such as CEP83, CEP164, or ANKRD26 between CAPture-MS and bead-only samples of Jurkat cells (Figure 5C, middle and bottom plots). The DAP component CEP89 was also reduced in Jurkat centrosomes, but not as much as other DAP proteins, confirming previous suggestions that CEP89 is also part of sDAPs.^56^ Overall, quantitative TMT analysis showed good agreement with CAPture-MS shotgun experiments, both demonstrating a specific reduction of DAP components in Jurkat centrosomes when compared with centrosomes of other cell types (Figures 5B and 6A).

### CAPture-MS confirms hierarchy of DAP assembly while revealing potential new components

DAP assembly begins by recruitment of a complex comprising MNR, OFD1, PIBF1/CEP90, and FOPNL, followed by CEP83 and downstream factors such as SCLT1, CEP164, FBF1, or ANKRD26.^2,57^ CAPture-MS indicates that Jurkat centrosomes lack all these factors except for MNR and FOPNL, a pattern that could be explained by absence of either OFD1 or PIBF1 in Jurkat centrosomes. Searching publicly available genomic data^58^ from Jurkat cells revealed potential frameshift mutations in *PIBF1* (*p.L495fs*) and *OFD1* (*p.K393fs*), of which the latter was confirmed in our cell line (Figure 6B). Whereas *PIBF1* is presumably expressed bi-allelically, due to *OFD1* being an X-linked gene and Jurkat a male-derived cell line, *p.K393fs* is expected to cause loss of full-length OFD1 protein in Jurkat cells. Indeed, absence of OFD1 and DAPs in Jurkat cells was confirmed by immunoblotting and IF (Figures 6C–6E and S6B–S6D).

**Figure 7:**
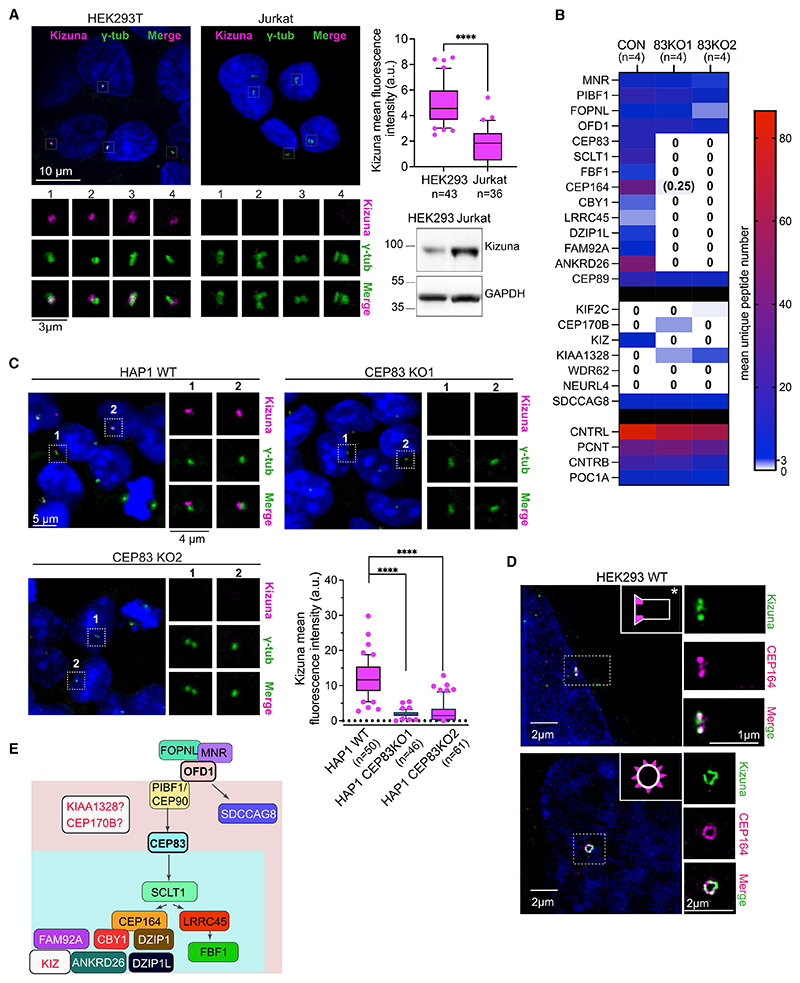
Combining gene editing with CAPture-MS identifies Kizuna (KIZ) as a DAP-associated component (A) Confocal micrographs of Kizuna localization in HEK293T and Jurkat cells. Cells were co-stained with γ-tubulin, a PCM marker (green). Higher magnification images of centrosomes are shown below. Box plots depict mean signal intensities of centrosomal Kizuna (Mann-Whitney, ****p < 0.0001). Boxes represent interquartile range with whiskers set at 10th and 90th percentiles. Western blot confirms expression of Kizuna in HEK293T and Jurkat cell lysates with GAPDH serving as loading control. (B) Heatmap shows the average number of unique peptides identified in CAPture-MS performed from control HAP1, CEP83-KO1, and CEP83-KO2 cells (n = 4 for each genotype). Top group: core DAP proteins and DAP-associated factors; middle group: proteins from pan-centrosome proteome that are not known to associate with DAPs but are absent from Jurkat centrosomes; bottom group: control group comprising centrosomal proteins unrelated to DAP. (C) Confocal micrographs of Kizuna localization in WT and CEP83-KO HAP1 cells. Cells were co-stained with γ-tubulin, a PCM marker. Boxplot depicts mean signal intensities of centrosomal Kizuna. Boxes represent interquartile range with whiskers set at 10th and 90th percentiles (Mann-Whitney, ****p < 0.0001). (D) Structured illumination microscopy (SIM) images of Kizuna localization in centrosomes of HEK293T cells. Kizuna (green) co-localizes with the DAP marker CEP164 (magenta). Cartoons illustrate longitudinal (top) and cross-section (bottom) centriole orientation. Corresponding high magnification images are shown on the right. In cartoon with asterisk, orientation is assumed as centriole proximal ends are not stained. (E) Cartoon of OFD1- and CEP83-dependent (beige and blue, respectively) DAP modules based on CAPture-MS analyses of Jurkat and HAP1 centrosomes. Putative new components are shown in white boxes.

Not all proteins absent from Jurkat centrosomes are known DAP components (e.g., Kizuna [KIZ], WDR62, and SDCCAG8), suggesting that OFD1 and/or DAP loss could impact centrosomal enrichment of additional factors (Figure 6F). While Kizuna protein is expressed in Jurkat cells, in IF, Jurkat centrosomes displayed either a faint diffuse Kizuna signal or no signal at all (Figure 7A). By contrast, a sharp centrosomal signal was detected in all HEK293T cells. To establish suitability of CAPture-MS for dissecting protein hierarchies in centrosomes and identify further DAP components, in HAP1 cells we genetically ablated CEP83, a DAP protein acting downstream of OFD1 (Figure S7). CAPture-MS from two CEP83-KO clones confirmed absence of SCLT1, CEP164, FBF1, and ANKRD26 along with the DAP-associated transition zone components CBY1, DZIP1, and FAM92A from centrosomes, but as expected, OFD1, FOPNL, MNR, and PIBF1/CEP90 remained centrosomal (Figure 7B; Table S7).

Similarly to Jurkat cells, Kizuna was absent from centrosomes of CEP83-KO but not WT HAP1 cells in CAPture-MS and IF (Figures 7B and 7C). In 3D structured illumination microscopy (SIM), Kizuna co-localizes with the DAP component, CEP164, at the distal end of centrioles in HEK293T centrosomes (Figure 7D). These results imply that Kizuna is a DAP-associated protein. Mutations both in *CEP164* and Kizuna are associated with retinal degeneration and dystrophy,^59,60^ suggesting that these two components may play a common role in disease pathogenesis. However, despite overall peptide numbers being lower in these HAP1 samples (Table S7) compared with other HAP1 datasets (Table S3), Kizuna was the only pan-centrosome protein that was missing from both CEP83-KO HAP1 and Jurkat centrosomes. Since SDCCAG8, CEP170B, KIF2C, and KIAA1328 were all identified in CEP83-KO HAP1 centrosomes (Figures 6F, 7B, and 7E), it will be interesting to see if these components require OFD1 for centrosomal accumulation or whether they are differentially expressed/localized between HAP1 and Jurkat cells. According to public databases SDCCAG8 is expressed in Jurkat cells,^58^ so its absence from centrosomes is most likely due to loss of OFD1; indeed, the two proteins interact.^61^

Altogether our results demonstrate the power and sensitivity of CAPture-MS to detect differences between centrosome proteomes, while raising the possibility that DAPs might be subject to cell-type- or disease-specific control.

## Discussion

Here, we demonstrated that CAPture combined with MS provides high-coverage centrosome and basal body proteomes with improved sensitivity and specificity over conventional SDG-based methods. As a high-throughput method CAPture is well suited to study changes in centrosome proteomes resulting from genetic alterations as well as those arising during dynamic processes such as cell-cycle progression or ciliogenesis.

We showed that Ninein is essential for, whereas the CEP128 contributes to, CAPture, indicating that the CAPture peptide is likely to interact with sDAPs in a Ninein-dependent manner. Furthermore, since efficacy of CAPture from mitotic cells (where Ninein is exclusively at the PCM) was far greater than that from CEP128-KO cells (Figures 3F and 3G), CEP128 contributes to binding between the CAPture peptide and Ninein complexes either directly (i.e., conformation) or indirectly (i.e., centrosomal recruitment). The exact binding partner of the peptide remains unclear but given the requirement for Ninein, it may be Ninein or its interactor, CEP170. CEP170 is a strong candidate as it localizes to sDAPs and proximal centriole ends in a Ninein-dependent manner and was previously shown to interact with CCDC61.^43–45^ Importantly, however, the CAPture peptide does not appear to interact with soluble cytoplasmic pools of either CEP128, CEP170, or Ninein even when centrioles are absent because all three proteins were significantly reduced in CAPture-MS of centrinone-treated cells when compared with control (Figure 2C).

During development of the CAPture method, we found sonication and salt concentration to be particularly critical for efficient centrosome isolation. We have not explored the full range of buffer and detergent options, and it is therefore feasible that its performance can be further improved. While CAPture worked well from several cell types, it largely failed in HeLa and HepG2 cells. There could be multiple reasons for the differential performance. First, access of the CAPture peptide to its binding target may differ between cell types. Indeed, the importance of salt concentration for CAPture suggests that the epitope for the peptide-centrosome interaction needs unmasking. Second, centrosomes are low-abundance organelles, and therefore, CAPture-MS works best when background protein levels are low. Therefore, detection of centrosome proteins may be further improved by blocking free streptavidin with biotin following conjugation of CAPture peptide.

Being a highly sensitive and specific method, CAPture-MS is uniquely suitable to dissect hierarchies of centrosomal protein complexes and uncover protein dependencies as demonstrated by our discovery of Kizuna as a DAP-associated protein. By accurately highlighting differences between centrosome proteomes, CAPture-MS revealed loss of DAPs in centrosomes of the widely used T lymphocyte line, Jurkat, which we showed was due to a deleterious frameshift mutation in *OFD1*. Although lymphocytes are not ciliated,^62^ our results highlight the vulnerability of the *OFD1* locus and hence the ciliogenesis pathway in other cell types in cancer. Due to *OFD1* being an X-linked gene, male cancer cells can be rendered cilia-less by a single damaging point mutation in *OFD1*, potentially increasing their proliferative capacity. Even when OFD1 is unaltered, its expression and therefore DAP/cilia formation could be dampened by X chromosome silencing, which occurs due to erroneous XIST expression in some male cancer cells.^63^ Nonetheless damaging frameshift mutations in *OFD1* have been detected in 29 cell lines^58^ (depmap.org) and several human cancers (cBioPortal).^64^ A recent study on the genomic landscape of cancers^65^ revealed reduced overall survival in patients with altered OFD1.

In summary, we developed a centrosome affinity purification method, CAPture, and demonstrated its broad utility for proteomic and structural studies of centrosomes and cilia. CAPture constitutes a unique toolkit to explore centrosome proteome dynamics in health and disease and further improvement of this methodology could expand the number of cell types and tissues it can be utilized on. A particularly exciting direction is combining proximity-labeling approaches with CAPture for high-resolution spatial mapping of centrosomal protein networks.

### Limitations of the study

Our study has a number of limitations. First, the precise binding mechanism between the CAPture peptide and centrosomes remains to be established. Although our data show a requirement for Ninein, we cannot rule out contributions from other factors such as sonicated chromatin, properties of the magnetic beads, or charge distribution of the CAPture peptide. Second, as we have already highlighted CAPture is not effective in every mammalian cell line or tissue, thus hindering comprehensive analysis of centrosome proteomes across all cell types and tissues. With better insight into the molecular interaction between the CAPture peptide and centrosomes, it might become possible to predict performance of CAPture in a particular cell type. Lastly, CAPture works best in a high-salt concentration lysis buffer, which is likely to cause loss of certain PCM proteins including more loosely associated enzymatic regulators. To combat this, it will be important to explore alternative buffers, detergents, and cell lysis methods.

## Star★Methods

## Key Resources Table

**Table:** 

REAGENT or RESOURCE	SOURCE	IDENTIFIER
Antibodies		
Rabbit polyclonal ANKRD26	GeneTex	Cat#GTX128255; RRID: AB_2885741
Rabbit polyclonal ARL13B	Proteintech	Cat#17711-1-AP; RRID: AB_2060867
Rabbit polyclonal CP110	Proteintech	Cat#12780-1-AP; RRID: AB_10638480
Rabbit polyclonal CEP164	Proteintech	Cat#22227-1-AP; RRID: AB_2651175
Mouse monoclonal CEP164	SantaCruz	Cat#sc-515403
Rabbit polyclonal CEP170	Abcam	Cat#ab72505; RRID: AB_1268101
Mouse monoclonal CNTROB	Abnova	Cat#H00116840-B01P; RRID: AB_1555868
Rabbit polyclonal FBF1	Atlas	Cat#HPA023677; RRID: AB_1848440
Rabbit polyclonal OFD1	Proteintech	Cat#22851-1-AP; RRID: AB_2879177
Mouse monoclonal γ-tubulin	Sigma	Cat#T9026; RRID: AB_477593
Rabbit polyclonal CP110	Proteintech	Cat#12780-1-AP; RRID: AB_10638480
Rabbit polyclonal CDK5RAP2	Produced in Gergely lab	N/A
Rabbit polyclonal CEP41	Bethyl	Cat#A301-798; RRID: AB_2780131
Rabbit polyclonal CEP83	Proteintech	Cat#26013-1-AP; RRID: AB_2880334
Rabbit polyclonal CEP128	Atlas	Cat#HPA001116; RRID: AB_1078323
Rabbit polyclonal CEP152	Bethyl	Cat#A302-479A; RRID: AB_1966085
Mouse monoclonal CETN3	Abnova	Cat#H00001070-M01; RRID: AB_464016
Rabbit polyclonal CRBN	Proteintech	Cat#11435-1-AP; RRID: AB_2085739
Rabbit polyclonal DDB1	Proteintech	Cat#11380-1-AP; RRID: AB_2088808
Rabbit polyclonal EDC4	Proteintech	Cat#17737-1-AP; RRID: AB_10665813
Mouse monoclonal NIN	Proteintech	Cat#67132-1-Ig; RRID: AB_2882431
Mouse monoclonal NIN	Santa Cruz	Cat#sc-376420; RRID: AB_11151570
Rabbit polyclonal NOP53	Proteintech	Cat#27353-1-AP; RRID: AB_2880852
Rabbit polyclonal PLK1	Novus	Cat#NB100-547; RRID: AB_10002724
Rabbit polyclonal PCNT	Abcam	Cat#ab4448; RRID: AB_304461
Rabbit polyclonal TRIM27	Proteintech	Cat#12205-1-AP; RRID: AB_2256660
Mouse monoclonal g-tubulin	Sigma	Cat#T6557; RRID: AB_477584
Mouse monoclonal SAS6	Santa Cruz	Cat#sc-81431; RRID: AB_1128357
Donkey polyclonal anti-Goat IgG (H+L) Cross- Adsorbed Secondary Antibody, Alexa Fluor 488	Life Technologies	Cat#A-11055; RRID: AB_2534102
Donkey polyclonal anti-Mouse IgG (H+L) Highly Cross-Adsorbed Secondary Antibody, Alexa Fluor 594	Life Technologies	Cat#A-21203; RRID: AB_141633
Polyclonal Donkey anti-Mouse IgG (H+L) Highly Cross-Adsorbed Secondary Antibody, Alexa Fluor 488	Life Technologies	Cat#A-21202; RRID: AB_141607
Donkey polyclonal anti-Rabbit IgG (H+L) Highly Cross-Adsorbed Secondary Antibody, Alexa Fluor 594	Life Technologies	Cat#A-21207; RRID: AB_141637
Donkey polyclonal anti-Mouse IgG (H+L) Highly Cross-Adsorbed Secondary Antibody, Alexa Fluor 647	Life Technologies	Cat#A-31571; RRID: AB_162542
Donkey polyclonal anti-Rabbit IgG (H+L) Highly Cross-Adsorbed Secondary Antibody, Alexa Fluor 647	Life Technologies	Cat#A-31573; RRID: AB_2536183
Amersham ECL Mouse IgG, HRP-linked whole Ab	Cytiva	Cat#NXA931-1ML; RRID: AB_772209
Donkey Anti-Rabbit IgG, Whole Ab ECL Antibody, HRP Conjugated	Cytiva	Cytiva Cat# NA934, RRID:AB_772206
Bacterial and virus strains		
Escherichia coli DH5a	Thermo	EC0112
C41(DE3)	Cambridge Bioscience	60442
Chemicals, peptides, and recombinant proteins		
Biotinylated CCDC61 peptide (Biotin- SPSPTGGRALRFDPTAFVKAKERKQREIQMKQQ)	Biomatik	N/A
Dynabeads™ M-280 Streptavidin	ThermoFisher Scientific	Cat# 11205D
cOmplete™, Mini, EDTA-free Protease Inhibitor Cocktail	Roche	Cat# 11836170001
10 nm gold	BBI Solutions	Cat# EM.GC10/4
5 ml HiTrap Q HP column	Cytiva	Cat# 17115401
Recombinant DNA		
pLipo-3xFLAG-CCDC61 ^334-366^	This paper	N/A
Deposited data		
Proteomic dataset 1 (Tables S1, S2, S3, S4, and S5)	This paper	PRIDE: PXD040308
Proteomic dataset 2 (Table S6)	This paper	PRIDE: PXD040309
Proteomic dataset 3 (Table S5)	This paper	PRIDE: PXD043906
Proteomic dataset 4 (Table S7)	This paper	PRIDE: PXD043692
Experimental models: Cell lines		
HEK293 CEP128KO	Produced in Gergely lab	N/A
HAP1 NIN-KO and CEP83-KO	Produced in van Breugel lab	N/A
Jurkat E6.1	ATCC	RRID: CVCL_0367
U2OS	ATCC	RRID: CVCL_0042
HEK293	ATCC	RRID: CVCL_0045
Raji	ATCC	RRID: CVCL_0511
HAP1	Horizon Discovery	RRID: CVCL_Y019
FS13b	Cambridge BRC hIPSCs core facility	N/A
U-251MG	ATCC	RRID: CVCL_0021
hTERT-RPE1	ATCC	RRID:CVCL_4388
Oligonucleotides		
EDC4 CAGGUACAGCGCAUCGUUAtt	Ambion, Silencer Select	#1; s24265
EDC4 CCUGUUCUGUGACAACCAUtt	Ambion, Silencer Select	#2; s24266
TRIM27 CAAAAAUGUCUAUUCUUGAtt	Ambion, Silencer Select	#1; s11960
TRIM27 GCUGAACUCUUGAGCCUAAtt	Ambion, Silencer Select	#2; s11959
NOP53 GAACCAAAGUCCAGAAGAAtt	Ambion, Silencer Select	#1; s26871
NOP53 CUUCGAGACCGGUUCAAGAtt	Ambion, Silencer Select	#2; s26873
CCDC171 GGAGAAGCAUUGCGACAAAtt	Ambion, Silencer Select	#1; s47495
CCDC171 GGUCUGCAAAUGCAAUUAAtt	Ambion, Silencer Select	#2; s47493
CEP128gRNA_1 GTCGTGACCGATTGAGTCCA	Sigma-Aldrich	gRNA
CEP83 gRNA_1 GGCTGAAGTAGCGGAATTAA	Sigma-Aldrich	gRNA
CEP83 gRNA 2 TAATTTACGGGCAGAACGTT	Sigma-Aldrich	gRNA
Ninein gRNA 1 CTGGAAGACGCAACGCAGTG	Sigma-Aldrich	gRNA
CEP83_clone1_forward CCTTGTATGCTTTCTTTAAA ATTATTATTAGATTGCAAGACTGGAGGAAGATAAAG	Sigma-Aldrich	primer
CEP83_clone1_reverse GCTTGGGGGGCATACA TTTATGCTCTAAAAATCAAAGGCTTGTC	Sigma-Aldrich	primer
CEP83_clone2_forward GAAAAATGATGAATTTTAAA CTATTTTGTTTGTATGAAAGGCTGAAAAACAATC	Sigma-Aldrich	primer
CEP83_clone2_reverse CTAAAGAATGAAACAGAAAC ATAAAACAACTTACTTTACTGGACAATGTATTTATTTCTCG	Sigma-Aldrich	primer
Ninein_forward GCACACTAATCTTCTCTTGCCTTCT CTAGCACTG	Sigma-Aldrich	primer
Ninein_reverse CTTCCAGGCACGTCCTGACACACTC	Sigma-Aldrich	primer
CEP128_forward 1 CTGTGTGGCCTTTACCTGTG	Sigma-Aldrich	primer
CEP128_reverse 1 TTGAGACCCAGTGAGACCAG	Sigma-Aldrich	primer
CEP128_forward 2 AGCAGAGACAATGGAGGAGG	Sigma-Aldrich	primer
CEP128_reverse 2 GGCAGCCTCTAGAAACCAGA	Sigma-Aldrich	primer
Softwares and algorithms		
Prism v9.5.1	Graphpad Software LLC	N/A
Chromagnon091Mac	https://doi.org/10.1038/nprot.2017.020 ^67^	N/A
ImageJ 2.9.0/1.53t	https://imagej.net/downloads	N/A
SerialEM	https://doi.org/10.1016/j.jsb.2005.07.007 ^70^	N/A
IMOD	https://doi.org/10.1006/jsbi.1996.0013 ^71^	N/A
NovaCTF	https://doi.org/10.1016/j.jsb.2017.07.007 ^72^	N/A
Others		
Carbon Film 400 Mesh, Cu	Electron Microscopy Sciences	Cat# CF400-Cu-50
Quantifoil™ R 2/1 on 200 copper mesh	Quantifoil	Cat# X-102-Cu200

## Resource Availability

### Lead contact

Further information and requests for resources and reagents should be directed to and will be fulfilled by the lead contact, Fanni Gergely (fanni.gergely@bioch.ox.ac.uk).

### Materials availability

HAP1 NIN KO cells, HEK293 CEP128 KO cells and HAP1 CEP83-KO cells will be available upon request by emailing the lead contact.

## Experimental Model And Study Participant Details

### Cell lines and culture

HEK 293T/17 (HEK 293T) cells were grown in Dulbecco’s Modified Eagle’s Medium (DMEM) (Gibco) supplemented with 10% heat-inactivated Fetal Bovine Serum (hi-FBS) (Gibco). U251 and U2OS cells were grown in DMEM GlutaMAX (Gibco) supplemented with 10% hi-FBS. FreeStyle 293-F cells (kindly donated by Dr Roger Williams, MRC Laboratory of Molecular Biology) were grown in FreeStyle 293 Expression Medium (ThermoFisher) in a shaker at 37°C, 8% CO2 and 125 rpm. Jurkat clone E6-1 (Jurkat) cells were grown in suspension in RPMI-1640 medium (Gibco) supplemented with 10% hi-FBS (Gibco). Patient-derived primary glioblastoma cell line G7^66^ (kindly donated by Dr Harry Bulstrode, MRC Cambridge Stem Cell Institute), were grown in DMEM/HAMS-F12 (Gibco) supplemented with 0.14 % D-(+)-Glucose solution (10%; Sigma-Aldrich), 1 X MEM NEAA (100 X; Gibco), 0.01% Bovine Albumin Fraction V (7.5 %; Gibco), 0.1 mM 2-Mercaptoethanol (50 mM; Gibco), 0.5 X B27 supplement (50 X, serum-free; Gibco) and 0.5 X N2 supplement (100 X; Gibco), on dishes pre-coated with 50 μg/ml Laminin (Cultrex). N1E-115 cells were grown in DMEM High Glucose (Gibco) supplemented with 10% hi-FBS (Gibco). hTERT RPE-1 (RPE-1) cell lines were grown in DMEM F-12 GlutaMAX (Gibco) supplemented with 10% hi-FBS (Gibco). HAP1 cells were grown in IMDM-GlutaMAX supplemented with 10% hi-FBS (Gibco). The human induced pluripotent stem cell (hiPSC) line, FS13B, was cultured in TesR™-E8™ medium (STEMCELL) in 6-well plates precoated with 10 μg/ml Vitronectin XF™ (STEMCELL). Media was exchanged daily and colonies were allowed to grow between 5 and 7 days before passaging. All adherent cells were cultured at 37°C in a humidified chamber with 5% CO_2_. Cell line identities of HAP1, U251, HEK293T and RPE1 were confirmed with STR genotyping.

## Method Details

### Drug treatments

Before isolation of centrosomes by the sucrose sedimentation-based technique (see Section 4.2.2), HEK 293T cells were treated with 1 μg/ml nocodazole (Sigma-Aldrich) and 1 μg/ml cytochalasin-B (Alfa Aesar) for 1 hours at 37 °C in5 % CO_2_. For centrinone treatments, HEK 293T cells were treated with 150 nM Centrinone (Tocris) for a period of 8 days, with passaging of cells and fresh drug supplemented every 2-3 days. For cell cycle synchronisation, HEK 293T cells were treated with 2.5 mM Thymidine (Sigma-Aldrich) for a period of 18 hours or with 100ng/ml nocodazole for 20 hours at 37 °C in 5 % CO_2_.

### Cell cycle analysis by flow cytometry in HAP1 and HEK293T cells

HAP1 cells were grown to 50% confluence in a T-75 flask. Hoechst 33342 (EMP Biotech, cat. number F-0409-M005.0-001) was added to the media at the final concentration of 2 μM and incubated at 37°C with 5% CO2 for 90 min. Cells were analysed by flow cytometry using an iCyt EC800 cell analyser (Sony Biotechnology) and the resulting cell-cycle distribution was determined using FCS EXPRESS 6 Flow software (De Novo Software). For HEK293T cells, propidium iodide was used at 20 μg/ml to stain nuclei and cells were analysed in BD FACSAria Fusion cell analyser. Plots were generated by FloJo V10.9.0.

### siRNA transfections

siRNA sequences are listed in the key resources table. siRNA transfections were carried out using Lipofectamine RNAiMAX (Invitrogen), according to the manufacturer’s instructions. The final siRNA final concentration was 50 nM. Cells were processed for downstream analysis 48 hours after transfection. As a negative control, a non-targeting siRNA from Ambion (siRNA ID: 4390084, Silencer Select) was used in all the experiments.

### Tissue preparation

All animal procedures were performed in accordance with the Animal Welfare and Ethical Body of the CRUK Cambridge Institute (CRUK CI, University of Cambridge), and UK Home Office regulations (in accordance with UK law, Animals Scientific Procedures Act 1986). Mice were housed under specific pathogen-free conditions and cared for in the CRUK CI Biological Resource Unit. Mice used in this study were of the C57BL/6 background.

Whole livers and spleens were isolated from 10-week-old female C57BL/6 mice. Cells were isolated from the liver using a cell strainer (Easystrainer 70 μm, greiner bio-one); liver pieces were passed through the strainer using PBS and the back of a syringe, after which cells were pelleted at 300 g for 5 mins. Supernatant (containing fat) was discarded and pellet was washed with PBS and spun again. The final pellet was resuspended in Buffer P1 (see section 3.5) and lysed for 45 minutes on rollers at 4 °C. Cells were isolated from the spleen by placing the whole spleen in a 15 ml tube containing Buffer P1 and lysed using the TissueRuptor (Qiagen, USA) for approximately 30 seconds, until tissue was broken up. Thereafter, cells were lysed for 45 minutes on rollers at 4 °C.

### Imaging

#### Immunofluorescence

Cells were seeded and grown on glass coverslips (VWR) and fixed with 100 % ice-cold (-20 °C) methanol for spectroscopy (ACROS Organics) for 5 minutes at - 20 °C. After fixation, cells were permeabilised in PBST (PBS, 0.5 % (v/v) Tween 20 (Promega)), or for centriolar marker staining, in the extraction buffer (0.5 % (v/v) Triton X-100 (ACROS Organics)), 0.05 % (w/v) sodium dodecyl sulfate (SDS) (Sigma-Aldrich) and 0.5 % (v/v) Tween 20 (Promega) in PBS) for 5 minutes at room temperature. Thereafter, cells were blocked in 5 % (w/v) Bovine Serum Albumin (BSA) (Sigma-Aldrich) in PBS for 15 minutes at room temperature, or overnight at 4 °C. Coverslips were incubated with primary antibodies diluted in 5 % BSA in PBS according for 2 hours at 37 °C. The following antibodies were used for immunofluorescence (source and dilution factors are stated, see also key resources table): ANKRD26 (GeneTex; 1:500); ARL13B (Proteintech; 1:300); CP110 (Proteintech; 1:250); CEP83 (Proteintech; 1:300); CEP128 (Atlas, 1:200); CEP152 (Bethyl, 1:500); CEP164 (Proteintech; 1:400); CEP170 (Abcam; 1:200); CETN3 (Abnova, 1:300); CNTROB (Abnova; 1:200); EDC4 (Cell Signaling; 1:200) FBF1 (Atlas; 1:100); NIN (Proteintech; 1:300); OFD1 (Proteintech; 1:300); PCNT (Abcam; 1:500); SAS6 (Santa Cruz; 1:500); SCLT1 (Atlas; 1:50); TRIM27 (Proteintech; 1:200) and γ-tubulin (Sigma; 1:300).

Following 4 washes coverslips were incubated with secondary antibody conjugated to Alexa Flour 488 or 555 (Invitrogen) in 5 % BSA in PBS, for 1 hour at 37 °C in the dark. Coverslips were then washed before incubation with 1 μg/ml Hoechst 33258 (Sigma-Aldrich) in PBS, to visualize DNA and were mounted on glass slides (SuperFrost Ultra Plus, Thermo Scientific) using the ProLong Diamond Antifade Mountant (Invitrogen), and dried overnight at room temperature before being stored at 4 °C.

#### Confocal microscopy

Confocal images of fixed cells were taken using the Confocal White Light Laser (WLL) Leica TCS SP8 Microscope or a Zeiss LSM780 inverted microscope (100x objectives).

All the images were acquired as z-stacks (0.3 or 1 μm step size). Images were taken using the HC Plan Apo 100 x/1.40 OIL (CS2) objective and image acquisition was carried out with the Leica Application Suite X (LAS X) software (Leica Microsystems). For higher magnification images of centrosomes on coverslips, an optical zoom of 12 was applied, and single-focal plane images were acquired. These images were then exported to Hyugens Professional (Scientific Volume Imaging), and the Express Deconvolution tool was used for deconvolution of images, with no changes to manufacturers settings. After acquisition, all images acquired were imported into Fiji (version: 2.0.0-rc-59/1.51k) to obtain maximum intensity projections unless otherwise stated. The images were then converted to RGB and saved as TIFF files. All image quantification was performed in Fiji.

#### 3D-SIM

Super-resolution 3D-SIM imaging was performed on a DeltaVision OMX SR microscope system (GE Healthcare) equipped with an Olympus UPlanApo 60x 1.5 NA oil immersion objective, pco.edge sCMOS cameras and 405, 488, 561 and 640 nm lasers. Image stacks were acquired with a z-distance of 125 nm and with 15 raw images per plane (5 phases, 3 angles). Spherical aberration was corrected by using the objective’s correction collar. Raw datasets were reconstructed with softWoRx 6.5.2 (GE Healthcare) using channel-specific measured optical transfer functions (OTFs) and a Wiener filter setting of 0.0020 to achieve a lateral (x-y) resolution of ~110 nm and an axial (z) resolution of approximately ~280 nm. Image data quality was assessed using SIMcheck before applying the auto-thresholding and 16-bit conversion utility of SIMcheck. Colour channels were 3D-registered using Chromagnon 0.85 using a reference image of EdU-labelled nuclei.^67^

### Cell lysis and western blotting

Cells were lysed in RIPA buffer (50 mM Tris-HCl pH 8.0, 150 mM NaCl, 1 mM EDTA, 1 % (v/v) NP-40, 0.5 % (w/v) Na-deoxycholate, 0.1 % (w/v) SDS), supplemented with protease inhibitor cocktail tablets (Complete EDTA-free, Roche Diagnostics)). After incubating the samples on ice for 45 minutes, cell lysates were centrifuged at 14000 g for 15 minutes at 4 °C. Supernatants (cytoplasmic fraction) were transferred to new 1.5 ml tubes and the pelleted nuclear fractions were discarded. The protein concentration of the extracts was determined using the Direct Detect system (Millipore), after which protein samples were mixed with NuPAGE LDS Sample Buffer (Invitrogen), supplemented with NuPAGE Sample Reducing Agent (Invitrogen), and heated at 80 °C for 10 minutes. Protein extracts were separated on a Bolt 4-12 % Bis-Tris Plus gel (Invitrogen) in 1xMOPS buffer (Invitrogen) and transferred from the SDS polyacrylamide gel onto nitrocellulose membrane (0.45μm). Following appropriate washes, membrane was incubated with primary and then horseradish peroxidase (HRP)-conjugated secondary antibodies diluted in 5 % milk in TBST. Pierce ECL Western Blotting Substrate (Thermo Scientific) was used for detection according to the manufacturer’s instructions. The following antibodies were used for immunoblotting (source and dilution factors are stated, see also key resources table): CP110 (Proteintech; 1:500); CDK5RAP2 (Produced in Gergely lab, 1:500); CEP41 (Bethyl; 1:1000); CEP83 (Proteintech; 1:1000); CEP83 (Sigma; 1:500); CEP128 (Atlas; 1:500); CEP152 (Bethyl; 1:500); CETN3 (Abnova; 1:500); CRBN (Proteintech; 1:1000); DDB1 (Proteintech; 1:1000); EDC4 (Cell Signaling; 1:1000); NIN (Proteintech; 1:1000); NIN (Santa Cruz; 1:500); NOP53 (Proteintech; 1:500); PLK1 (Novus; 1:500); PCNT (Abcam; 1:2000); SAS6 (Santa Cruz; 1:500); TRIM27 (Proteintech; 1:1000) and γ-tubulin (Sigma; 1:500).

### Centrosome affinity purification by CAPture

CAPture was based on a biotinylated peptide pulldown described previously.^68^ Centrosome isolation was performed using streptavidin-coated magnetic beads (Dynabeads™ M-280 Streptavidin; Invitrogen) and a biotinylated peptide corresponding to a 33 amino acid fragment of CCDC61 (Biotin-SPSPTGGRALRFDPTAFVKAKERKQREIQMKQQ; synthesized >95% purify in Biomatik), which was dissolved in DMSO or TBS to make 5 mg/ml stock peptide solution. The leftover stock peptide was snap-frozen and stored in -80 °C. Briefly, beads (60 μl per pulldown) were washed 3 times in TBS-N (TBS with 0.1 %(v/v) NP-40) and once in CAPture buffer (50 mM Tris-HCl pH 8.0, 300 mM NaCl, 0.2 %(v/v) NP-40, 10 %(v/v) glycerol, protease inhibitor cocktail (Complete EDTA-free, Roche Diagnostics) and phosphatase inhibitor cocktail (PhosStop; Roche Diagnostics)). Beads were then resuspended in CAPture buffer and peptide (6 μl per pulldown) was added, before being placed on a rotating wheel at 12 rpm for 1.5 hours at 4 °C. Cells (3 x 10^7^ per pulldown) were collected in PBS and lysed in CAPture buffer for 30 minutes on ice, sonicated briefly (700W ultrasonic processor, 2 x 3 second pulses, 30% amplitude, 3mm microtip probe (418-21; Fischer Scientific)) and centrifuged at 1800 g for 10 minutes at 4 °C. Supernatants were incubated with beads only or beads plus peptide (pre-washed four times in CAPture buffer before being resuspended in a suitable volume of CAPture buffer) on a rotating wheel at 12 rpm for 2 hours at 4 °C. After incubation, beads were washed four times with CAPture buffer. Samples for western blots were resuspend in 2xNuPAGE LDS Sample Buffer (Invitrogen), supplemented with NuPAGE Reducing Agent (Invitrogen), and then boiled at 80 °C for 10 minutes. Thereafter, beads were removed from the sample using a DynaMag™-2 magnet (Invitrogen). For SDS-PAGE, unless otherwise indicated 1.5% of the total cell lysate was loaded, whereas bound lane represents 30-100% bead-bound protein. Proteins were detected using standard ECL substrate, with exposure times of no longer than 3 minutes. Samples for mass spectrometry were washed twice in 100 mM Ammonium Bicarbonate (AMBIC; Fisher Chemical), before being flash-frozen in dry ice and stored at -80°C. For analysis of purified centrosomes by immunofluorescence microscopy, beads collected by DynaMag™-2 magnet were resuspended in CAPture buffer after the final wash, pipetted onto 1.5 mg/ml Poly-L-lysine (Sigma-Aldrich)-coated coverslips (Precision 1.5H, Marienfeld Superior) in a 24-well plate and spun at 2500 g for 10 minutes at 4 °C.

### Protein purification

3xFLAG-CCDC61^334-366^ was cloned in a lipoyl-tag *E. coli* expression vector.^36^ The plasmid was transformed into C41(DE3) cells, which were grown in 2xYT media. Protein expression was induced by 1 mM IPTG at 16 °C overnight. The cells were collected by centrifugation, and their pellet snap-frozen in liquid nitrogen before resuspension in 50 mM Tris pH8.0, 300 ml NaCl, 2 mM 2-mercaptoethanol, 10 mM imidazole, 1 mM AEBSF. Cells were lysed by sonication, and the cell debris was removed by centrifugation. The supernatant was mixed with Ni-NTA beads and incubated on a rotating platform at 4 °C for 90 min. The beads were washed with 50 mM Tris-HCl pH7.4, 300 ml NaCl, 2 mM 2-mercaptoethanol, 30 mM imidazole, and bound proteins were eluted with 50 mM Tris-HCl pH7.4, 300 ml NaCl, 2 mM 2-mercaptoethanol, 300 mM imidazole. The lipoyl tag was digested from 3xFLAG-CCDC61^334-366^ by overnight dialysis against 30 mM Tris pH8.0, 150 ml NaCl, 2 mM 2-mercaptoethanol, 20 mM imidazole at 4°C in the presence of TEV protease, and subsequently removed by binding to Ni-NTA beads. 3xFLAG-CCDC61^334-366^ was diluted 2-fold with 20 mM Tris-HCl pH7.4, 2 mM DTT and loaded onto a 5 ml HiTrap Q HP column (Cytiva) equilibrated with 10 mM Tris-HCl pH7.4, 100 mM NaCl, 2 mM DTT. Bound proteins on the column were eluted using a linear salt gradient to 10 mM Tris-HCl pH7.4, 1 M NaCl, 2 mM DTT. The first peak fractions were collected, of which NaCl concentration was calculated from the elution profile and adjusted to ~300 mM. The collected fractions were concentrated to ~3 mM 3xFLAG-CCDC61^334-366^ before being snap-frozen in liquid nitrogen and storage at -80 °C.

### Electron microscopy of centrosomes and basal bodies

Centrosomes/basal bodies bound on the CAPture beads were eluted with the CCDC61 peptide fused to 3xFLAG tag as follows. 6 μl of 100 mM Bicine pH9.0 and 2.5 μl of 5 M NaCl were added to 30 μl of 3 mM 3xFLAG-CCDC61^334-366^. 30 μl of this elution peptide mix were added a 1.5 ml tube containing 500 μg of the M280 Dynabeads that captured centrosomes/basal bodies as described above. The beads were left at RT for 30 min while they were gently suspended by flicking the tube occasionally. The supernatant containing centrosomes/basal bodies was collected by placing the tube on a magnetic stand.

For negative-stain EM, 3 μl of the eluted centrosomes were applied once for Figure 1E and three times for Figures 3B and S2C toa 400-mesh carbon-coated cupper grid, which was grow discharged. The grid was washed with water twice and was incubated with 5 μl of 2%(w/v) phosphotungstic acid pH7.0 for 1 min before excess of the stain was removed using filter paper. Figure 1E were collected using the FEI Tecnai T12 microscope equipped with Gatan Ultrascan 1000 at MRC LMB whereas Figures 3B and S2C were collected using the FEI Tecnai F20 equipped with FEI CETA at the Astbury Biostructure Laboratory in the University of Leeds.

For cryo-ET, 6 μl of 10 nm gold (BBI Solutions) were added to 30 μl of the eluted centrosomes/ basal bodies. 3.5 μl of the mix were applied to a 200-mesh holey carbon R2/1 copper grid (Quantifoil), which was glow discharged for 45 sec at 25 mA. A Vitrobot (FEI), which was operated at 22 °C and 100% humidity, was used to remove the liquid excess by filter paper and vitrify the grid by plunging it into liquid ethane. Micrographs were collected using a FEI Titan Krios 2 microscope equipped with a Gatan K2 Summit direct electron detector with energy filter (20 eV slit width) at MRC LMB. The Volta phase plate^69^ was inserted to enhance contrast of the micrographs. Tilt series were collected using SerialEM^70^ at 33,000x nominal magnification (3.7 A° / pixel) on the counting mode and at 3° increments with the dose-symmetric scheme between -60° and 60° tilt angles. The defocus was set at -0.75 μm. 10 movie frames per tilt angle were collected with dose of 0.25 e^-^/Å/frame. Alignframes in Etomo^71^ (as used to align movie frames. Tomogram reconstruction was performed using Etomo from IMOD using weighted backprojection. Contrast transfer function estimation and correction were performed using NovaCTF.^72^

### Centrosome isolation by sucrose gradient centrifugation

Centrosomes were purified based on the protocol by Moudjou and Bornens^22^ with minor modifications.^73^ Briefly, HEK 293T cells were treated with 1 μg/ml nocodazole and 1 μg/ml cytochalasin-B for 1 hour, washed in ice-cold PBS, before scraping and centrifugation at 1200 g. The pellet was then resuspended in 25 ml ice-cold TBS, centrifuged at 1200 g for 5 minutes and resuspended in 25 ml cold 8 % sucrose-0.1 % TBS. Following centrifugation at 1000 g for 5 minutes at 4 °C, the pellet was lysed in 45 ml Lysis Buffer (1 mM Tris HCl pH 8.0, 0.5 % NP-40, 0.5 mM MgCl_2_, 0.1 % β-mercaptoethanol (added fresh before lysis), protease inhibitor cocktail (Complete EDTA-free, Roche Diagnostics) and phosphatase inhibitor cocktail (PhosStop, Roche Diagnostics)). Lysates were centrifuged at 1800 g for 10 minutes at 4 °C and the supernatant was filtered through a 70 μm cell-strainer cap (BD Falcon), after which 1 M K-PIPES pH 7.2, 1 mM EDTA, and 2250 U DNaseI (Sigma-Aldrich) was added to achieve a pH of 6.8, and supernatants were incubated on ice for 15 minutes. Supernatants were pre-fractionated on a 50 % (w/w) sucrose cushion at 11000 rpm for 30 minutes at 4 °C using the SW32 rotor (Beckman Coulter), and then separated on a discontinuous sucrose gradient for 2 hours at 25000 rpm using the SW40 Ti rotor (Beckman Coulter). Sucrose solutions were prepared in 10 mM K-PIPES pH 7.2, 1 mM EDTA, 0.1 % β-mercaptoethanol (freshly added), 0.1 %Triton-X100 (freshly added), protease inhibitor cocktail (Complete EDTA-free, Roche Diagnostics) and phosphatase inhibitor cocktail (PhosStop, Roche Diagnostics)). For the discontinuous sucrose gradient, we layered 2 ml 70 % (w/w) sucrose solution, 1.5 ml 50% (w/w) sucrose solution, and 1.5 ml 40% (w/w) sucrose solution on top of each other in Ultra-Clear tubes (14 x 95 mm, Beckman). After centrifugation, 0.5 ml sucrose fractions were collected from the top of the gradient with a p1000 pipette and transferred into separate polycarbonate tubes (11 x 34 mm, Beckman). 800 μl K-PIPES pH 7.2 was added to each collected fraction, and tubes were centrifuged at 35000 rpm for 20 minutes 4 °C using the MLA-130 rotor (Beckman Coulter). Pellets were either resuspended in 10 mM K-PIPES pH 7.2 and prepared for western blotting or flash-frozen in dry ice and stored at -80°C for MS analysis.

### Mass spectrometry

All mass spectrometry (MS)-based experiments were carried out by the Proteomics Core Facility at the Cancer Research UK Cambridge Institute except for CAPture-MS of RPE1 and CEP83-KO cells, which were performed at the University of Leeds and MRC-LMB, respectively.

#### “On bead” tryptic digestion of proteins

Streptadividin beads with CAPture peptide and centrosomes or without (bead-only) were trypsinised with 100 ng of trypsin in 100 mM Ammonium Bicarbonate (AMBIC, Sigma) for overnight at 37 °C. Trypsin was then added for a further 4 hours at 37 °C. The supernatant was then collected and the reaction stopped with 0.5 % (v/v) formic acid (FA). Peptides were desalted using C18 cartridges (Biochrom) that were conditioned and equilibrated with 50 % acetonitrile (Fisher Scientific) and 0.1 % FA, respectively. Peptide-loaded cartridges were washed with 0.1 % FA and eluted with 60 % acetonitrile/0.1 % formic acid. Dried peptides were reconstituted in 0.1 % FA acid, for further LC–MS/MS analysis.

For CEP83-KO experiments, streptadividin beads with CAPture peptide and centrosomes were reduced with 5 mM DTT at 56 °C for 30 min and alkylated with 10 mM iodoacetamide in the dark at room temperature for 30 min. Samples were incubated with Lyc_C protease (Promega) for 4 h at 37°C, followed by overnight digestion with trypsin (Promega). Supernatants were transferred to fresh tubes, beads were washed once with 50% acetonitrile/ 0.1% TFA and washes were combined with the corresponding supernatants. Samples were then acidified, speed vac (Savant) to remove acetonitrile and desalted using home-made C18 stage tips (3M Empore) packed with oligo R3 (Thermo Scientific) resin. Bound peptides were eluted with 30-80% acetonitrile in 0.5% FA and partially dried down in Speed Vac.

For RPE1 cells, streptadividin beads with CAPture peptide and centrosomes were suspended in 4x LDS sample buffer (ThermoFisher) and taken for S-TRAP™ digestion, as per the instructions (PROTIFI, NY, USA). Eluted peptides were concentrated in the speedvac concentrator and reconstituted in 0.1% FA.

#### “In solution” tryptic digestion of proteins (sucrose sedimentation samples)

Sucrose fractionated pellets were resuspended in 20 μl lysis buffer containing 100 mM Triethylammonium bicarbonate (TEAB, Sigma), 0.1 % SDS (Sigma) followed by heating at 90 °C for 5 minutes and probe sonication (Active motif). Complete samples were reduced with 2 μl 50 mM tris-2-caraboxymethyl phosphine (TCEP, Sigma) for 1 hour at 60 °C followed by alkylation with 1 μl 200 mM methyl methanethiosulfonate (MMTS, Sigma) for 10 minutes at RT. Protein samples were then digested overnight at 37 °C using trypsin (Thermo Scientific) solution at ratio protein:trypsin ~ 1:30. The next day, protein digest was acidified with FA to a final concentration of 0.5 % (v/v). The peptides were desalted using C18 cartridges (Biochrom) as mentioned above. Dried peptides were reconstituted in 0.1% FA acid, for further LC–MS/MS analysis.

#### Liquid chromatography tandem mass spectrometry (LC-MS/MS)

Unless specified all experiments were run on either a QExactive or QExactiveHF mass spectrometer both coupled to an Ultimate 3000 RSLCnano HPLC system (Dionex). In both cases the following settings were used. Dried peptide samples were reconstituted in 25 μl of 0.1 % (v/v) FA, and 5 μl injected into the LC column for analysis (for sucrose sedimentation samples, each dried peptide sample was reconstituted in 10 μl of 0.1 % (v/v) FA, and 10 μl injected into the LC column for analysis). Peptides were loaded and separated on a reverse-phase trap column (length: 2 cm, inner diameter; 100 μm) and analytical column (length: 25 cm, inner diameter: 75 μm), respectively with 5–45 % acetonitrile gradient in 0.1 % FA at 300 nl/min flow rate. In each data collection cycle, one full MS scan (400–1,600 m/z) was acquired in the Orbitrap (60K resolution, automatic gain control (AGC) setting of 3x10^6^ and Maximum Injection Time (MIT) of 100 ms). The most abundant ions with a top 10 setting were selected for fragmentation by High-energy Collision induced dissociation (HCD). HCD was performed with a collision energy of 28 %, an AGC setting of 2x1010^4^, an isolation window of 2.0 Da, a MIT of 100 ms. Previously analysed precursor ions were dynamically excluded for 30 s.

For CAPture-MS of CEP83-KO experiments, samples were analysed by LC-MS/MS using a Q Exactive Plus hybrid quadrupole-Orbitrap mass spectrometer (Thermo Fisher Scientific) coupled on-line to a fully automated Ultimate 3000 RSLC nano System (Thermo Scientific). Peptides were trapped by a 100 μm x 2 cm PepMap100 C18 nano trap column and separated on a 75μm× 25 cm, nanoEase M/Z HSS C18 T3 column (Waters) using a binary gradient consisting of buffer A (2% acetonitrile, 0.1% FA) and buffer B (80% acetonitrile, 0.1% FA) at a flow rate of 300 nl/min. The mass spectrometer was operated in DDA mode, performed full-scan MS1, at m/z = 380-1600 with a resolution of 70K, followed by MS2 acquisitions of the 15 most intense ions with a resolution of 17.5K. NCE of 27% and isolation window =1.5 m/z were used. Dynamic exclusion was set for 40s.

For RPE1 samples, LC-MS/MS analyses of peptide mixtures were done using Vanquish Neo UHPLC system connected to Orbitrap Eclipse Tribrid mass spectrometer (Thermo Fisher Scientific). Prior to LC separation, tryptic digests were online concentrated and desalted using a trapping column (300 μm × 5 mm, mPrecolumn, 5μm particles, Acclaim PepMap100 C18, Thermo Fisher Scientific) at room temperature. After washing of trapping column with 0.1 % formic acid (FA), the peptides were eluted (flow rate –0.25 nl/min) from the trapping column onto an analytical column (EASY spray column, Acclaim Pepmap100 C18, 2μm particles, 75 μm × 500 mm, Thermo Fisher Scientific) at 45 °C by a 120 min linear gradient program (2-40 % of mobile phase B; mobile phase A: 0.1 % FA in water; mobile phase B: 0.1 % FA in 80 % ACN). Equilibration of the trapping column and the analytical column was done prior to sample injection to the sample loop. The analytical column with the emitter was directly connected to the ion source. MS data were acquired in a data-dependent strategy. The survey scan range was set to *m/z* 350-2000 with the resolution of 120,000 (at *m/z* 200) with a target value of 3×10^6^ ions and maximum injection time of 50 ms. HCD MS/MS (30% normalized fragmentation energy) spectra were acquired for maximum injection time of 54 ms and resolution of 30 000 (at *m/z* 200). Dynamic exclusion was enabled for 60 s. The isolation window for MS/MS fragmentation was set to 1.2 *m/z*.

#### Tandem Mass Tag (TMT) quantitative proteomics

Streptadividin beads with CAPture peptide and centrosomes or without (bead-only) were treated as in *“On bead” tryptic digestion of proteins*. Following elution with 60% acetonitrile/0.1% FA, peptides were labelled with the TMT-10plex plus reagents (Thermo Scientific) according to manufacturers’ instructions for 1 hour. All the samples were mixed and dried with speed vac concentrator. The TMT-mix samples were fractionated with Reversed-Phase cartridges at high pH (Pierce #84868). Nine fractions were collected using different elution solutions in the range of 5–50 % ACN as per manufacturers protocol. Dried peptides were reconstituted in 0.1% FA, for further LC–MS/MS analysis. Peptide fractions were analysed on a Dionex Ultimate 3000 system coupled with the nano-ESI source Fusion Lumos Orbitrap Mass Spectrometer (Thermo Scientific). Peptides were trapped on a 100 μm ID X 2 cm microcapillary C18 column (5 μm, 100 A) followed by 2-hour elution using 75 μm ID X 25 cm C18 RP column (3 μm, 100 A) with 5–45 % acetonitrile gradient in 0.1 % FA at 300 nl/min flow rate. In each data collection cycle, one full MS scan (380–1,500 m/z) was acquired in the Orbitrap (120K resolution, automatic gain control (AGC) setting of 3x10^5^ and Maximum Injection Time (MIT) of 100 ms). The subsequent MS2 was conducted with a top speed approach using a 3 second duration. The most abundant ions were selected for fragmentation by collision induced dissociation (CID). CID was performed with a collision energy of 35 %, an AGC setting of 1x1010^4^, an isolation window of 0.7 Da, a MIT of 35 ms. Previously analysed precursor ions were dynamically excluded for 45 seconds. During the MS3 analyses for TMT quantification, precursor ion selection was based on the previous MS2 scan and isolated using a 2.0 Da m/z window. MS2–MS3 was conducted using sequential precursor selection (SPS) methodology with the top10 settings. HCD was used for MS3, performed using 55 % collision energy and reporter ions were detected using the Orbitrap (50K resolution, an AGC setting of 5x1010^4^ and MIT of 86 ms).

### CRISPR/Cas9 genome editing

#### Ninein and CEP83 knockout HAP1

guide RNAs (gRNAs) targeting *H. sapiens CEP83* (NCBI NG.051825.1) and *NINEIN* gene (NCBI NG.032968.1) were designed by CRISPRdirect (https://crispr.dbcls.jp; Naito et al.^74^) and cloned into the CRISPR Nuclease Vector available in the GeneArt® CRISPR Nuclease Vector Kit (ThermoFisher, cat. number A21174). gRNAs sequences are listed in the key resources table.

Plasmids were transfected into HAP1 cells using FuGENE® HD transfection reagent according to the manufacturer’s protocol (Promega). After 24 hours, single cells positive for orange fluorescence protein were sorted using a FACS Synergy system (Sony) into 3X96-well plates. Untransfected cells were single sorted as a negative control. Putative knockout cell lines were selected for absence of protein expression by Western blot using anti-CEP83 (Sigma) and anti-NINEIN antibodies (Santa Cruz Biotechnology). Two independent clones were selected for further characterization. Small cells were sorted using a FACS Synergy system (Sony) in 3X96-well plates (one cell per well) and transferred to a T-75 flask after 80% confluence was reached. Cells were frozen down and kept in liquid Nitrogen for long-term storage.

For DNA sequencing, cells were grown to 50% confluence in one well of a 96-well plate, washed 3X with PBS and lysed using Direct PCR Lysis reagent (Viagen Biotech, cat. number 301-C) supplemented with Proteinase K (ThermoFisher, cat. Number EO0491) at 5 μg/ml final concentration according to the manufacturer’s protocol. The PAM sequence containing region of the extracted genomic DNA was amplified by PCR using Q5® High-Fidelity DNA polymerase master mix (NEB, cat. Number M0492S) and the primer pairs as depicted in the key resources table. PCR products were sub-cloned into pJET1.2/blunt plasmid using CloneJET PCR Cloning Kit (ThermoFishcer, cat. number K1231) and transformed into chemically competent *E. coli* DH5α. QIAprep Spin Miniprep Kit (QIAGEN, cat. number 27104) was used for plasmid extraction and the plasmids were submitted to Sanger sequencing by Eurofins Genomics. Sequencing primers pJet1-FP (5’-ACTACTCGATGAGTTTTCGG-3’) and pJet1-RP (5’-TGAGGTGGTTAGCATAGTTC-3’) are available from GATC/Eurofins.

#### CEP128 knockout HEK293T

gRNA targeting *H. sapiens CEP128* was selected using CRISPR Design (www.crsipr.mit.edu), CRISPR Search (www.sanger.ac.uk/hgt/wge/find_crisprs), and ChopChop (www.chopchop.cbu.uib.no). gRNAs sequences are listed in the key resources table. Oligonucleotides were designed with overhangs containing the Bbs1 restriction site. The oligonucleotide pairs for CEP128 gRNA (sense and antisense) were phosphorylated using the T4 Polynucleotide Kinase (NEB), according to the manufacturer’s instructions, and annealed. The oligonucleotide duplexes were ligated into the pX458 plasmid (#48138, Addgene) previously digested with the BbsI restriction enzyme (NEB). Correct insertion of the gRNAs into pX458 was verified using sequencing. FuGENE HD (Promega) was used to transfect the gRNA-Cas9 pX458 vectors into HEK293T cells, according to the manufacturer’s instructions. 24 hours after transfection, GFP-positive cells were single sorted into 96 well plates by FACS, using BD FACSAria IIU (BD Biosciences). Untransfected cells were single sorted as a negative control. Single clones were expanded and screened by western blotting to identify putative CEP128 knock-out clones. Putative hits were subjected to genomic DNA sequencing (primers are listed in STAR Methods and Table S5). PCR products were cloned into the pJET1.2/blunt vector and 10 single bacterial colonies per clone were analysed using Sanger sequencing.

## Quantification And Statistical Analysis

### Immunofluorescence image quantification and analysis

For all the imaging data analyses, ImageJ/Fiji was used to quantify fluorescence signal. Data were then plotted on GraphPad Prism 9.0 (GraphPad) and appropriate statistical analyses were performed. Specific statistical tests are indicated in figure legends.

### Mass spectrometry data analysis

Proteome Discoverer 2.1 software (Thermo Scientific) was used for the processing of HCD tandem mass spectra. Spectra were searched against the Uniprot Homo sapiens or Mus musculus FASTA database using SequestHT. All searches were performed with a static modification of Methylthio at Cysteines (+45.988 Da). Methionine oxidation (+15.9949 Da) and Deamidation on Asparagine and Glutamine (+0.984 Da) were included as dynamic modifications. Mass spectra were searched using precursor ion tolerance 20 ppm (or 10ppm for RPE1) and fragment ion tolerance 0.02 Da. For peptide confidence, 1 % FDR was applied and peptides uniquely matched to a protein were used for further analysis. Figures and tables depict number of unique peptides, percentage protein coverage and protein scores. The latter is the sum of the scores of the individual peptide Xcorr values above the specified score threshold. SEQUEST search algorithm was used for which the score threshold is calculated as follows: 0.8+peptide_charge × peptide_relevance_factor where peptide relevance factor is a parameter with a default value of 0.4.

For CEP83KO cells, data from LC-MS/MS were processed using MaxQuant^75^ with the integrated Andromeda search engine (v.1.6.3.3). Cysteines carbamidomethylation was set as a fixed modification, while oxidation of methionine, acetylation of protein N-terminal and glutamine to pyro-Glu were set as variable modifications. Tryptic digestion up to 2 missed cleavages were allowed. Protein quantification requirements were set at 1 unique and razor peptide.

### Label-free quantification of centrosomes isolated from control or centrinone-treated HEK293T cells

HCD tandem mass spectra were processed with SequestHT on Proteome Discoverer 2.2 software. The node for SequestHT included the following parameters: Precursor Mass Tolerance 20ppm, Maximum Missed Cleavages sites 2, Fragment Mass Tolerance 0.02Da and Dynamic Modifications were Oxidation of M (+15.995 Da) and Deamidation of N, Q (+0.984 Da). The Minora Feature Detector node was used for label-free quantification and the consensus workflow included the Feature Mapper and the Precursor Ion Quantifier nodes using intensity for the precursor quantification. For peptide confidence, 1% FDR was applied and peptides uniquely matched to a protein were used for quantification.

Data processing, normalization, and statistical analysis of all datasets were carried out using the workflow based on qPLEXanalyzer^76^ package from Bioconductor. In LFQ analysis only peptides present in 2 out of 3 replicates of CAPture pulldown from control cells were kept. Those peptides with missing values in centrinone-treated samples were replaced by minimum value within the group. The remaining missing values were imputed with knn algorithm. The peptide values are summed to protein intensities without any normalization. The differential analysis was first performed between CAPture and corresponding bead-only samples of control and centrinone-treated cells to get proteins enriched in CAPture. The final differential analysis between CAPture pulldowns from control and centrinone was then performed only on the above selected proteins. The differential analyses were carried out using the Limma method. Multiple testing correction of P-values was applied using the Benjamini-Hochberg method to control the FDR.

### Quantitative analysis of Jurkat and U251 centrosomes by TMT

The Proteome Discoverer 2.1 or 2.4 software (Thermo Scientific) was used for the processing of CID tandem mass spectra. Spectra were searched against the Uniprot Homo sapiens FASTA database (taxon ID 9606) using SequestHT. All searches were performed using a static modification TMT6plex (+229.163 Da) at any N-terminus and on lysines and Methylthio at Cysteines (+45.988 Da). Methionine oxidation (+15.9949 Da) and Deamidation on Asparagine and Glutamine (+0.984 Da) were included as dynamic modifications. Mass spectra were searched using precursor ion tolerance 20 ppm and fragment ion tolerance 0.5 Da. Decoy database search was employed to generate high peptide confidence (1% FDR) and for quantification, information calculated from reporter ion intensities of peptides uniquely matched to a protein were used.

Data processing, normalization, and statistical analysis of all datasets were carried out using the workflow based on qPLEXanalyzer^76^ package from Bioconductor. The data for Jurkat and U251 cell line were first analyzed separately. In both the datasets, peptide intensities were normalized using within group median scaling treating bead-only and peptide pulldown as separate groups. Protein level quantification was then obtained by the summation of the normalized peptide intensities. Thereafter, a statistical analysis of differentially regulated proteins was carried out using the Limma method providing us with the list of proteins significantly enriched in each cell line compared to bead-only.

The analysis was then performed on the combined dataset of Jurkat and U251 cell line using these selected proteins only. In addition, for each cell line only those peptides are selected that are found in at least half of the samples. The missing values were then imputed using knn algorithm. The datasets were then combined to select only those proteins identified in both cell lines. Finally, a statistical analysis of differentially regulated proteins between two cell lines was carried out using the Limma method. Multiple testing correction of P-values was applied using the Benjamini-Hochberg method to control the FDR. Clustering and heat map in Figure 5B were generated with https://software.broadinstitute.org/morpheus/. Volcano plots in Figure 5C were generated by https://huygens.science.uva.nl/VolcaNoseR2/.^77^

## Supplementary Material

Supplemental information can be found online at https://doi.org/10.1016/j.devcel.2023.09.008.

Table S1

Table S2

Table S3

Table S4

Table S5

Table S6

Table S7

## Data Availability

The mass spectrometry data associated with this publication have been deposited in the ProteomeXchange Consortium (http://proteomecentral.proteomexchange.org) via the PRIDE partner repository. Accession numbers can be found in the key resources table, whereas file names and descriptions are included in relevant Supplemental Tables. This paper does not report original code. Any additional information required to reanalyze the data reported in this work paper is available from the lead contact upon request.

## In brief

Centrosomes organize the microtubule cytoskeleton and play fundamental roles in animals. Here, Carden, Vitiello et al. report a simple, one-step centrosome purification method that allows studying the diversity of centrosome composition, structure, and function across different cell types and demonstrates its utility for centrosome research.

## Highlights

- CAPture enables affinity purification of mammalian centrosomes
- CAPture utilizes a peptide derived from CCDC61 to isolate centrosomes
- Combined with mass spectrometry CAPture delivers high-coverage proteomes
- CAPture will facilitate centrosome research in health and disease
